# DDR2 Coordinates EMT and Metabolic Reprogramming as a Shared Effector of FOXQ1 and SNAI1

**DOI:** 10.1158/2767-9764.CRC-22-0013

**Published:** 2022-11-09

**Authors:** Allison V. Mitchell, Jason Wu, Fanyan Meng, Lun Dong, C. James Block, Won-min Song, Bin Zhang, Jing Li, Guojun Wu

**Affiliations:** 1 Barbara Ann Karmanos Cancer Institute, Department of Oncology, Wayne State University School of Medicine, Detroit, Michigan.; 2 Department of Biology, Purdue University, West Lafayette, Indiana.; 3 Comprehensive Cancer Centre of Drum Tower Hospital, Medical School of Nanjing University and Clinical Cancer Institute of Nanjing University, Nanjing, P.R. China.; 4 Department of Breast Surgery, Qilu Hospital, Shandong University, Jinan, Shandong Province, P.R. China.; 5 Department of Genetics and Genomic Sciences, Icahn Institute of Genomics and Multiscale Biology, Icahn Mount Sinai School of Medicine, New York, New York.

## Abstract

While multiple transcription factors (TF) have been recognized to drive epithelial–mesenchymal transition (EMT) in cancer, their interdependence and context-dependent functions are poorly understood. In this study, we show that FOXQ1 and SNAI1 act as independent TFs within the EMT program with a shared ability to upregulate common EMT-TFs without reciprocally impacting the expression of one another. Despite this independence, human mammary epithelial cells with ectopic expression of either FOXQ1 or SNAI1 share a common gene set that is enriched for a DDR2 coexpression signature. Further analysis identified DDR2 as the most upregulated receptor tyrosine kinase and a shared downstream effector of FOXQ1 and SNAI1 in triple-negative breast cancer (TNBC) cell lines. Alteration of DDR2 expression in either FOXQ1- or SNAI1-driven EMT models or in TNBC cells resulted in a profound change of cell motility without significantly impacting EMT marker expression, cell morphology, or the stem cell population. Finally, we demonstrated that knockdown of DDR2 in the FOXQ1-driven EMT model and TNBC cell line significantly altered the global metabolic profile, including glutamine-glutamate and aspartic acid recycling.

**Significance:**

The critical role of DDR2 in cancer metastasis has been well established. However, the exact function of DDR2 in driving cancer metastatic progression remains unclear. The results of our current study provide new insights into the cancer-driving function of DDR2, suggesting that DDR2, as a shared effector of the EMT program, may drive tumor progression by promoting breast cancer cell motility and metabolic reprogramming.

## Introduction

The epithelial-to-mesenchymal transition (EMT) program is critical during embryogenesis and tissue repair. EMT reprogramming allows a polar, sedentary epithelial cell to acquire motility and plasticity of a mesenchymal cell (1–3). In cancer, EMT is thought to be initiated by the cross-talk of numerous signaling pathways within the tumor microenvironment. The core of EMT regulation is linked to a limited number of well-characterized transcription factors (TF). Members of the SNAI1 (Snail family transcriptional repressor), TWIST (Twist family BHLH transcription factor), ZEB (Zinc finger E-box-binding homeobox), and FOX (Forkhead box protein) families have been identified as the most critical EMT regulators (4–6). While these EMT-TFs exhibit distinct expression profiles, a complex regulatory network exists among them, and each of these TFs is sufficient to induce EMT in a tissue-specific manner (7, 8). Thus, EMT is now recognized as multiple, overlapping transcription programs, and a better understanding of the necessary components of EMT warrants further investigation.

The discoidin domain receptor (DDR) family of extracellular matrix (ECM) receptors has been associated with cancer and other diseases such as fibrosis, another aberrant form of EMT. The DDR family consists of two receptor tyrosine kinases (RTK), DDR1 and DDR2, that exhibit a unique pattern of delayed and sustained activation (9). DDR2 can be activated by either fibrillar or nonfibrillar collagen and is mainly expressed in fibroblastic cells (10–12). While DDR1 and DDR2 have been linked to cancer progression and metastasis, a downregulation of DDR1 and concomitant upregulation of DDR2 has been explicitly observed in EMT (13, 14). Consistent with this pioneering discovery, a DDR1^low^/DDR2^high^ protein profile has been associated with worse overall survival for triple-negative breast cancer (TNBC; ref. 15). DDR2 was shown to regulate SNAI1 stability through stimulating ERK2 activity and thereby facilitate breast cancer metastasis (16). DDR2 has been reported to be induced by TWIST1 in ovarian cancer (17). DDR2 has also been shown to mediate hypoxia-induced EMT in breast cancer cells (18). However, the exact role of DDR2 within an EMT program and metabolomic regulatory effects related to EMT have not been intensively investigated.

Accumulating evidence has found metabolic reprogramming capable of inducing EMT through multiple pathways, including glycolysis, the tricarboxylic acid (TCA) cycle, lipid, and amino acid metabolism. Reciprocally, metabolic dysregulation is further exacerbated by EMT-TFs (19, 20). This relationship between EMT and metabolic reprogramming has been intensively investigated. Previous metabolomic profiles have identified a common set of metabolites to be increased across multiple cell models of EMT, including glutamine, glutamate, beta-alanine, and the dipeptide glycyl leucine, representing a closely connected metabolic network of glutaminolysis, TCA, and pyrimidine metabolism (21). In line with this, EMT-TFs suppress or enhance the expression of metabolic enzymes in different biological contexts (22–28), and multiple glycolytic enzymes are also involved in EMT regulation (29–31). Moreover, mutations in the TCA cycle enzymes FH (fumarate hydratase), SDH (succinate dehydrogenase), and IDH (isocitrate dehydrogenase) were confirmed to induce EMT (32–34). Altogether, these results suggest a regulatory link between the EMT transcription program and metabolism that warrants further investigation in cancer progression.

In this project, we sought to characterize the EMT-TF network based on models established in human mammary luminal epithelial cells (HMLE) and in TNBC cell lines. We found that FOXQ1 (forkhead box Q1) and SNAI1 cannot reciprocally regulate each other but share a significant overlap in the downstream transcription program. These common FOXQ1-SNAI1 gene targets are enriched for the gene coexpression signatures of the RTKs, platelet-derived growth factor receptor β (PDGFRβ), and DDR2. Specifically, DDR2 was the most significantly upregulated RTK downstream of both SNAI1 and FOXQ1. Using FOXQ1- and SNAI1-driven EMT models and TNBC cells, we performed functional analysis to investigate the contribution of DDR2 to the EMT phenotype and oncogenic properties. While ectopic expression and knockdown of DDR2 did not alter classical EMT features, we observed a significant alteration of cell motility in an EMT cell model and multiple TNBC cell models. Finally, using targeted metabolomic profiling, we identified that DDR2 significantly contributes to changes in metabolic flux in an EMT cell model and a TNBC cell model.

## Materials and Methods

### Cell Culture

All human breast cancer cell lines were obtained from and characterized by cytogenetic analysis by ATCC. All cell lines were grown by ATCC recommendations. The human mammary epithelial cell line HMLE was obtained from Robert A. Weinberg's laboratory at MIT. HMLE was maintained in the culture as described previously (5). All original cell lines were authenticated upon receipt by comparing them with the original morphologic and growth characteristics. The mouse breast cancer cell lines 4T1, 4T07, 168FARN, and 67NR were originally generated at Karmanos (35). These cells were cultured in high glucose DMEM supplemented with 5% FBS, 5% NCS (Newborn Calf Serum), NEAA (Non-Essential Amino Acids), and antibiotics (100 U/mL penicillin and 100 μg/mL streptomycin). BT549 (NCI-DTP, catalog no. BT-549, RRID:CVCL_1092), MDA MB231 (RRID:CVCL_0062), and BT20 (RRID:CVCL_0178) cell lines were used for functional studies. Cell line identities were verified using the GenomeLab short tandem repeat analyses (Beckman Coulter). Cells were routinely stained by Hoechst 33342 to ensure no *Mycoplasma* contamination. Cells used for experiments were within 20 passages from obtaining.

### Generation of Stable Cell Models

Full-length *FOXQ1*, *SNAIL1*, *TWIST1*, *ZEB2*, and *DDR2* plasmids were purchased from Open Biosystems. These genes were subcloned into the pENTR vector (RRID:Addgene_149548) and transferred into a pLenti6 vector (BRID: Addgene_21691) via homologous recombination. The lentivirus for the full-length gene was then generated using the lentivirus-expression system (Invitrogen). In addition, a set of short hairpin RNA (shRNA) clones for *DDR2*, *FOXQ1*, or *SNAIL1* was purchased from Open Biosystems. The information for the effective shRNA is available in Supplementary Primers and shRNA information. The lentivirus for the shRNA was then generated using the Trans-Lentiviral packaging system (Addgene). The generated lentivirus was then used to infect the targeted model cells. Stable cells were generated after being selected with Blasticidin (10 μg /mL) for the overexpression model or puromycin (12 μg/mL; Invivogen) for the knockdown model.

### qRT-PCR

A total of 1 μg of RNA from each cell line was used to generate cDNA with random hexamer primers using the Superscript III first-strand synthesis system for RT-PCR (Invitrogen). qRT-PCR was done using the iQSYBR Green Supermix (Bio-Rad). A GAPDH primer set was used as an internal control. The sequences of qPCR primers for all tested genes are available in Supplementary Primers and shRNA information.

### Western Blotting and Antibodies

Cells were lysed in the presence of 50 mmol/L Tris, pH 7.5, 150 mmol/L NaCl, and 0.5% NP-40 on ice. A total of 30 or 50 μg of total protein from each sample was resolved on a 6%–10% Bis-Tris gel with Tris/glycine/SDS running buffer and transferred to nitrocellulose membranes (Bio-Rad Laboratories). The blots were then probed with various antibodies, including β-catenin (BD Biosciences, catalog no. 610153, RRID:AB_397554, 1;1,000), Fibronectin (BD Biosciences, catalog no. 610077, RRID:AB_2105706, 1:1,000), N-cadherin (BD Biosciences, catalog no. 610920, RRID:AB_2077527, 1:1,000), E-cadherin (BD Biosciences, catalog no. 610405, RRID:AB_397787, 1:1,000), Vimen-tin (Cell Signaling Technology, catalog no. 5741, RRID:AB_10695459, 1:1,000), PDGFRβ (Cell Signaling Technology, catalog no. 3169, RRID:AB_2162497, 1:1,000), FGFR (Cell Signaling Technology, catalog no. 9740, RRID:AB_11178519, 1:1,000), Ror2 (Thermo Fisher Scientific, catalog no. PA5-14727, RRID:AB_2180121, 1:1,000), SNAl1 (Proteintech, catalog no. 13099-1-AP, RRID:AB_2191756, 1:500), DDR2 (Cell Signaling Technology, catalog no. 12133, RRID:AB_2797825, 1:500), and V5 antibody (Thermo Fisher Scientific, catalog no. R960-25, RRID:AB_2556564, 1:2,000). FOXQ1 antibody was generated by our own laboratory (1:10,000). β-actin antibody (Santa Cruz Biotechnology, catalog no. sc-47778, RRID:AB_626632, 1:2,000) was used for loading control. Signal detection was performed using Pierce ECL Western Blotting Substrate kit (Thermo Fisher Scientific, 32106).

### Cell Proliferation and Drug Resistance Analysis

Cells were seeded in triplicate at the density of 2.0  ×  10^3^ per well in 96-well plates on day 0. Cell proliferation was measured with MTT (Tetrazolium dye reduction) assay on days 1, 3, 5, and 7. All these experiments were repeated at least two times. To test the cell response to chemotherapy, the cells were treated with 0–10 nmol/L paclitaxel (Pac) or 0–100 nmol/L doxorubicin (Dox) for 24 hours with different doses as indicated. After being cultured in a drug-free growth medium for another 24 hours, the surviving cells will be quantified or used for other oncogenic properties analysis.

### Cell Migration and Invasion

Cell migration and invasion assays were performed as described previously (36). Briefly, cell migration and invasion assays were performed using the 24-well control chamber and Matrigel invasion chamber, respectively, according to the manufacturer's instructions (BD Biosciences). All cell lines were seeded at 1.0  ×  10^4^ cells per chamber with a whole culture medium without FBS. Medium with 10% FBS was used as a chemoattractant. A total of 24 hours after seed, migratory and invading cells were fixed and stained with a Diff-Quik kit.

### Colony Formation Assay

The nontarget (NT) and DDR2 knockdown cell models established on the basis of HMLE/FOXQ1 and HMLE/Snail cells were seeded in 6-well plates with a density of 5,000 cells/well in the presence of blasticidin and puromycin (3 μg/mL, Invivogen) for 2–3 weeks. At the endpoint, the plates were stained with crystal violet. The number of surviving foci were counted.

### Tumorsphere Formation

The mammosphere formation assay was performed as previously described with minor modifications (37). A total of 10,000 cells were plated in a 6-well ultra-low attachment plate (Corning Inc.) and grown in a sphere formation medium. The sphere formation medium is a serum-free DMEM/F12 (1:1) medium supplemented with B27 (Invitrogen), 20 ng/mL EGF, 1 μg/mL hydrocortisone, 5 μg/mL insulin, and 5 μg/mL β-mercaptoethanol. A total of 1 mL of medium was added to each well every other day for 8 days. Images of mammospheres were recorded, and the number of mammospheres was manually counted on day 10. Experiments were performed in triplicate and repeated two times.

### Flow Cytometry

FACS analysis was performed as described before (37). Cells were harvested with trypsin treatment and washed with PBS containing 2% FBS and 2% BSA. For CD44/CD24 labeling, combinations of fluorochrome-conjugated mAbs obtained from BD Biosciences against human CD44 (FITC, catalog no. 555478) and CD24 (PE, catalog no. 555428) or their respective isotype controls were added to the cell suspension at concentrations recommended by the manufacturer and incubated at 4°C in the dark for 30 minutes. The labeled cells were washed in the wash buffer and then analyzed on a BD LSR II flow cytometer (BD Biosciences). At least 2 × 10^5^ cells were counted.

### Differential Gene Expression Analysis (RNA Sequencing and Microarray)

RNA was isolated from HMLE/FOXQ1 and HMLE/LACZ cells using the RNeasy Plus Mini Kit (QIAGEN). For each sample, 2 μg RNA with a 260/280 above 2.0 was processed for library construction and sequencing. Samples were run in duplicate. Library preparation and sequencing were performed at LC Sciences. Briefly, the RNA library was prepared from Poly-A selection and subsequent processing using the TruSeq Stranded mRNA kit (Illumina) according to the manufacturer's protocol. Sequencing was performed using the Illumina HiSeq 2000 platform with 100 bp paired-end reads. Paired-end reads were mapped to the hg19 human genome using Bowtie2 v2.2.9 (Bowtie 2, RRID:SCR_016368). The abundance was estimated using RSEM, and the differential expression analysis was done using EdgeR v3.12.1 (RRID:SCR_012802) in the Bioconductor package (RRID:SCR_006442). Differentially expressed genes with *P* < 0.05 were selected (Bayes *t* test with corrections for multiple comparisons). Microarray data for HMLE/SNAI1 and HMLE-Vector control were downloaded from the NCBI Gene Expression Omnibus (GEO) database (GSE143349). Differentially expression was performed on RMA (Robust Multichip Average) values from triplicate samples using the GEO2R (RRID:SCR_016569) and the limma (RRID:SCR_010943) package (R Bioconductor V 3.11). Differentially expressed genes were selected using a statical cutoff of *P* < 0.05 by the Benjamini–Hochberg method. Each HMLE/EMT-TF model was compared with the respective HMLE control cell lines to identify all genes with >|2|-fold change. The lists with differentially expressed genes were then compared with give rise to a common set of genes that are upregulated or downregulated in both HMLE/FOXQ1 and HMLE/SNAIL relative to the respective controls.

### Functional Annotation and Gene Set Enrichment Analysis

Functional annotation and enrichment analysis were conducted lists of differentially expressed genes by statical overrepresentation tests using the Enrichr web platform (https://maayanlab.cloud/Enrichr/) and using the gene set enrichment analysis (GSEA) open-access software (V 4.1.0). Gene set enrichment upregulated in both HMLE/FOXQ1 and HMLE/SNAIL models, relative to the controls, by functional class scoring methods using the GSEA open-access software (V 4.1.0). GSEA was conducted on the log_2_ expression values with values for HMLE/FOXQ1, and HMLE/SNAIL1 samples in class A and HMLE control samples in class B. Genes were ranked using the log_2_ ratio of classes. Analysis was run with 1,000 permutations and an FDR cutoff of 0.25.

### The Cancer Genome Atlas and METABRIC Data Analysis

Normalized Illumina HT12v3 mRNA microarray data were downloaded from the European Bioinformatics Institute (38). The normalized gene expressions were adjusted for confounding factors, including batch and age, by capturing residuals with intercepts from the linear regression model (gene expression ∼ α1batch + α2age + α0) by lm() function from R software (version 3.4.2). We gathered TNBC samples and performed Spearman correlation analysis between the normalized DDR2 expressions and critical pathways in TNBC, including RTK and ECM. We also performed a nonparametric Kruskal–Wallis test on normalized DDR2 expressions between receptor-positive and TNBC samples.

### Quenching of Metabolism and Metabolite Extraction

For cell metabolism analysis, HMLE/Lac Z and HMLE/FOXQ1 cells were cultured in low glucose (100 μmol/L) DMEM/F12 medium containing 2 mmol/L glutamine and 10% FBS. All cell lines were grown in 6-cm tissue culture dishes with four biological replicates, and all samples were harvested at 80% confluence. For metabolite extraction, the medium was removed, and cells were washed with ice-cold PBS twice, then flash frozen with liquid nitrogen, and 1 mL of ice-cold 80% methanol in each well. Cells were collected by scraping and stored at −80°C. The resulting mixture was centrifuged at 5,000 × *g* for 5 minutes, and the supernatant was moved to a new tube. The remaining pellet was reextracted twice more with 500 μL of 80:20 methanol: water at −80°C, and all the supernatants were combined with the original supernatant. A total of 10 μL of the extract were injected into each LC/MS-MS for separations.

### Targeted LC/MS

We will follow the standard protocol in Pharmacology Core at Karmanos Cancer Institute, as described previously (39). Briefly, metabolites in tested cell lines were quantitatively profiled using an LC/MS-MS–based targeted metabolomics platform, which consists of 254 metabolites involved in major human metabolic pathways. All LC/MS-MS analyses were performed on an AB SCIEX QTRAP 6500 LC/MS-MS system, consisting of a SHIMADZU Nexera ultra-high-performance liquid chromatography coupled with a triple quadrupole/linear ion trap mass spectrometer. Analyst 1.6 software was used for system control and data acquisition, and MultiQuant 3.0 software was used for data processing and quantitation.

### Data Collection and Processing

Metabolomics data analyses were performed using the MetaboAnalyst web-based statistical package (http://www.metaboanalyst.ca/; RRID:SCR_015539; ref. 39). Metabolites with >50% missing values were removed from the analysis, and the remaining missing values were replaced by the minimum value of a metabolite. In addition, metabolite signals were normalized to total protein levels within each cell line and log2 transformed, and then autoscaled (mean centered and divided by each metabolite's SD). If significant, a one-way ANOVA followed by Fisher least-significant differences *post hoc* analysis was performed to identify significantly changed metabolites among groups. Significantly changed metabolites were presented as a heat map and ranked in ascending order according to the FDR-adjusted *P* values from the ANOVA test. Overrepresentation analysis was conducted on statistically differential metabolites using hypergeometric testing. Pathway topology analysis was performed using Relative-Betweenness Centrality metrics.

### Statistical Analysis

A two-sided independent Student *t* test without equal variance assumption was performed to analyze the results of cell growth, mammosphere formation, cell migration, and invasion results. The drug response was assessed by interpolating a sigmoidal drug–response standard curve and compared by the extra sum-of-squares F test using GraphPad Prism 8 (RRID:SCR_002798).

### Data Availability

All data from The Cancer Genome Atlas (TCGA) and METABRIC were downloaded by either cBioPortal or Firehose. Microarray for HMLE/SNAI1 cells and matched HMLE control from Xiong and colleagues was obtained from the NCBI GEO database (GSE143349; ref. 40). The original RNA sequencing (RNA-seq) data for HMLE/FOXQ1 and HMLE/LacZ cells are deposited in the NCBI GEO dataset #GSE141293. The overlapping FOXQ1-SNAI1 gene set that was identified by comparing these two datasets is available in Extended Data 1. The normalized metabolite concentrations and statistical results for significantly altered metabolites associated with shRNA DDR2 knockdown in HMLE/FOXQ1 and BT549 cells located in Extended Data 2 and Extended Data 3, respectively. Commonly altered metabolites associated with DDR2 knockdown in both cell models can be found in Supplementary Table S1.

## Results

### FOXQ1 and SNAI1 are Independent EMT TFs

**FIGURE 1:**
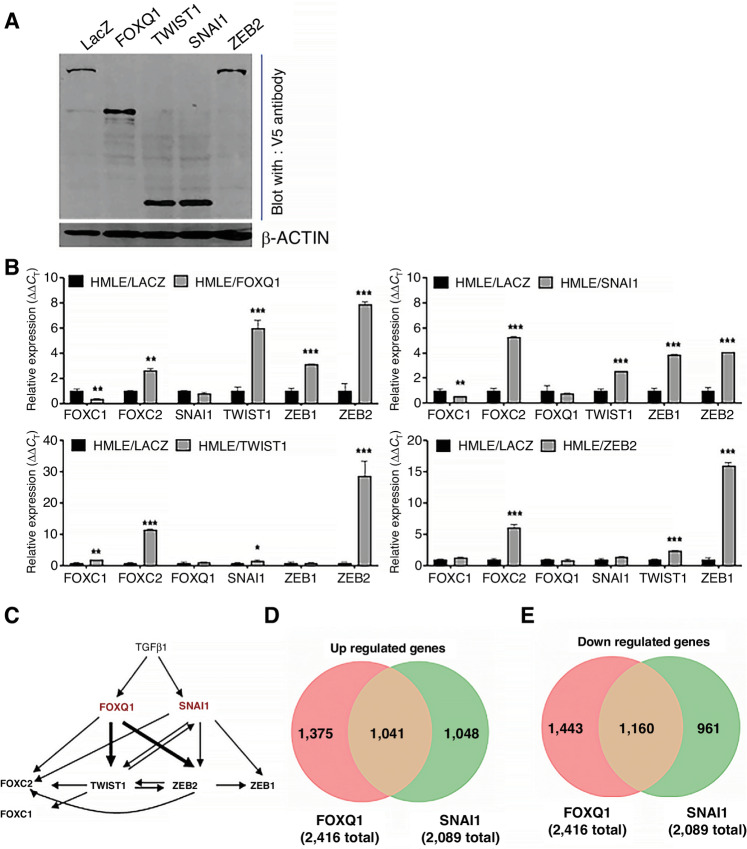
Identification of SNAI1 and FOXQ1 as independent EMT TFs. **A,** The exogenous expression level of each TF in the four EMT cell models was determined by Western blot analysis with an anti-V5 antibody. β-actin was used as a protein loading control. **B,** Expression of EMT-TFs in four different EMT cell models, including HMLE/FOXQ1, HMLE/TWIST1, HMLE/ZEB2, and HMLE/SNAI1. HMLE/LacZ served as a control. (*, *P* < 0.05; **, *P* < 0.01; and ***, *P* < 0.001). **C,** Summary of the regulatory relationship between core EMT programs. Venn diagram of the differentially upregulated (**D**) and downregulated (**E**) genes identified in HMLE/FOXQ1 and HMLE/SNAI1 cells, relative to the HMLE/LacZ control counterparts.

To uncover the regulatory relationship within the EMT-TF network, we performed qRT-PCR of four stable human mammary epithelial cell lines (HMLE) with ectopic *FOXQ1*, *SNAI1*, *TWIST1*, or *ZEB2* expression (Fig. 1A; ref. 41). Consistent with previous reports (42), we found that overexpression of an individual EMT-TF induces the expression of a select set of other EMT-TFs. TWIST1 upregulated the expression of *FOXC2* and *ZEB2*, and marginally increased *SNAIL1* and *FOXC1* expression. In comparison, ZEB2 upregulated *FOXC2*, *TWIST1*, and *ZEB1*. We found that SNAI1 upregulated *FOXC2*, *TWIST1*, *ZEB1*, and *ZEB2* expressions. Similarly, we also observed that FOXQ1 upregulated the expression of *FOXC2*, *TWIST1*, *ZEB1*, and *ZEB2*. FOXC1 was slightly downregulated in both the HMLE/FOXQ1 and HMLE/SNAI1 models. Interestingly, FOXQ1 and SNAI1 were not observed to regulate the expression of one another. However, they displayed the ability to regulate the expression of all other common EMT-TFs tested (Fig. 1B). As FOXQ1 and SNAI1 are both reported downstream targets of TGFβ signaling (43–45), these results suggest FOXQ1 and SNAI1 form two independent, parallel transcriptional axes within the TGFβ-controlled EMT program (Fig. 1C).

To identify the downstream effectors of the FOXQ1 and SNAI1 EMT programs, we compared the gene expression profiles of HMLE/FOXQ1 and HMLE/SNAI1 cell models. We performed paired-end RNA-seq of HMLE/FOXQ1 cells and matched HMLE/LacZ vector control for differential gene expression analysis. These results were compared with the differentially expressed genes in HMLE/SNAI1 and matched HMLE control cells obtained from a published microarray dataset (GSE143349; ref. 40). Differential expression analysis was performed for both HMLE/FOXQ1 and HMLE/SNAI1 samples relative to each control cell line to identify differentially upregulated or downregulated genes with *P* value < 0.05 and >|2|-fold change.

While we had found that FOXQ1 and SNAI1 function independently within the EMT-TF network (Fig. 1B and C), roughly 50% of the differentially expressed genes overlap within the two cell models. A total of 1,041 upregulated genes and 1,160 genes downregulated were commonly dysregulated between HMLE/FOXQ1 and HMLE/SNAI1 relative to control HMLE cells (Fig. 1D and E, and Extended Data 1). We first interrogated the biological functions unique to the transcription programs regulated by FOXQ1 or SNAI1 by the hypergeometric test. Examination of the 1,048 genes that are uniquely upregulated in HMLE/SNAI1 cells identified enrichment of processes associated with cell adhesion and migration (Supplementary Fig. S1A). Conversely, the 1,375 genes uniquely upregulated in HMLE/FOXQ1 cells had enrichment in ribosomal and translational processes (Supplementary Fig. S1B). Furthermore, we found that the top signaling pathways in the *SNAIL1* gene set included angiogenesis and cadherin signaling, while the *FOXQ1* gene set was enriched for regulation of apoptosis, insulin signaling, and hypoxia (Supplementary Fig. S1C and S1D). We similarly interrogated the functions of the genes that were uniquely downregulated in the respective cell models. The 961 genes that were downregulated explicitly in HMLE/SNAI1 cells were enriched for DNA repair and replication functions and signaling pathways related to immune cell function (T- and B-cell activation), epithelial signaling pathways (endothelial signaling, EGF), and apoptosis (Supplementary Fig. S1E and S1F). The 1,443 genes that were uniquely downregulated in HMLE/FOXQ1 cells shared similar functions to their upregulated counterparts, such as enrichment in the regulation of RNA processes, translation, and ribosome functions (Supplementary Fig. S1G). Moreover, the FOXQ1-specific downregulated gene set was additionally enriched for immune signaling pathways (Toll receptor and IL signaling), asparagine/aspartate metabolism, and the ubiquitin proteasome pathway (Supplementary Fig. S1H). Altogether, these data suggest that SNAI1 and FOXQ1 could regulate several distinct components of the EMT process.

### DDR2 is a Commonly Regulated RTK by EMT Program

**FIGURE 2:**
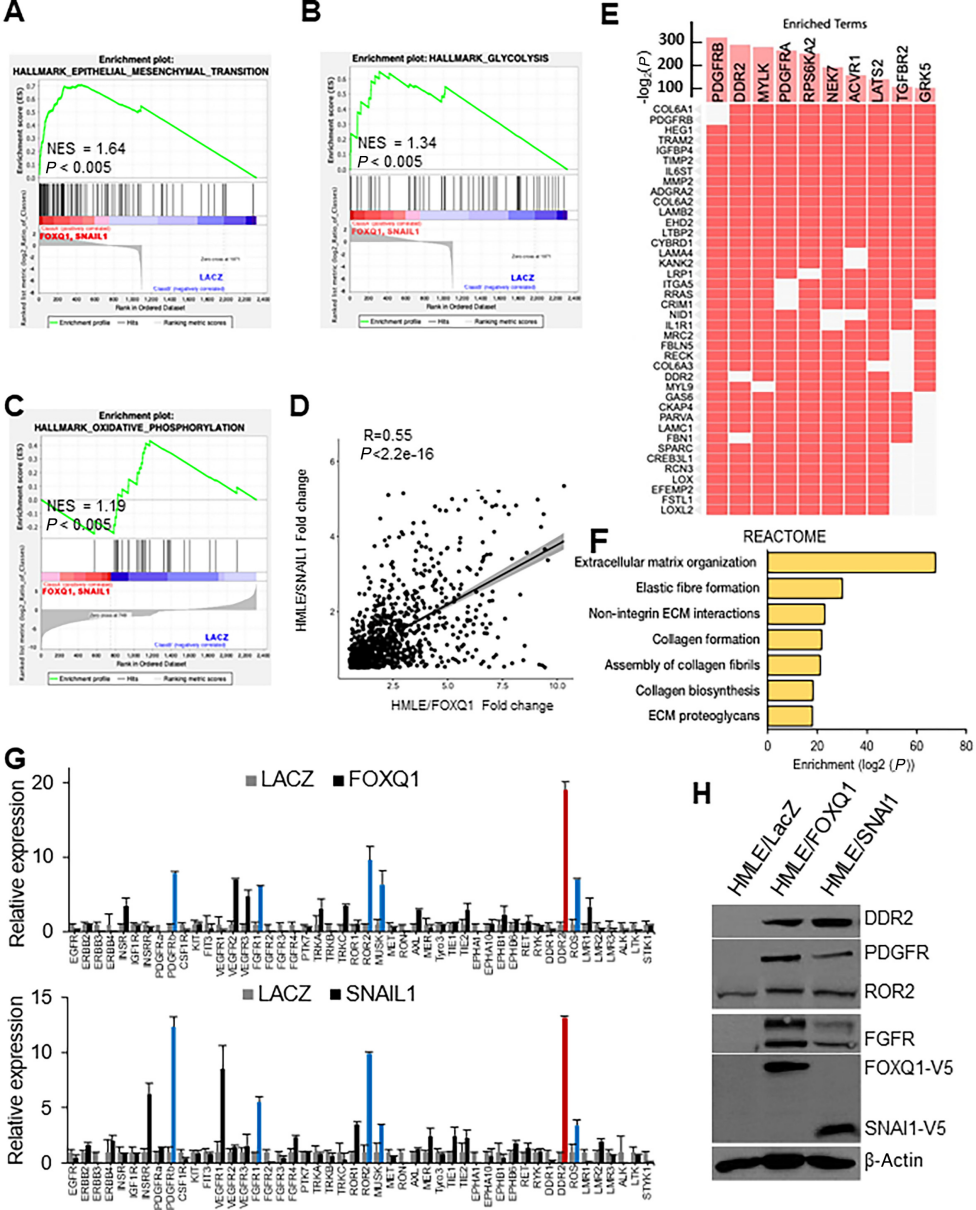
DDR2 is a commonly regulated RTK by FOXQ1 and SNAI1-controlled EMT programs. **A–C,** GSEA of the 2,201 genes commonly dysregulated in HMLE/FOXQ1, and HMLE/SNAI1 cells (both upregulated and downregulated) reveals enrichment of hallmark EMT functions (**A**) and glycolysis (**B**). LacZ control cells exhibited enrichment for oxidative phosphorylation (**C**). Class 1 represents HMLE/FOXQ1 and HMLE/SNAI1 samples, which class 2 represents HMLE/LacZ samples. **D,** Plot of the log2 fold change in expression for HMLE/FOXQ1 and HMLE/SNAI1 cells, relative to respective HMLE/LacZ control counterparts. Spearman correlation coefficient is reported. **E,** The FOXQ1-SNAI1 1,042 upregulated gene signature was assessed for the RTK coexpression signature from the ARCHS4 database. **F,** REACTOME pathway enrichment analysis of the 1,042 genes that are commonly upregulated in HMLE/FOXQ1 and HMLE/SNAI1 cell models**. G,** The expression profile of 48 RTKs by qRT-PCR identified a set of RTKs commonly regulated by FOXQ1 and SNAI1 in the respective HMLE models. **H,** Western blot analysis shows several RTKs were upregulated in HMLE cells with ectopic expression of FOXQ1 and SNAI1. β-actin was used as a protein loading control.

We next sought to characterize the overlapping functions within the FOXQ1 and SNAI1 transcriptome of HMLE cells. We performed GSEA of the 2,201 genes commonly dysregulated in HMLE/FOXQ1 and HMLE/SNAI1 cells. We observed an enrichment of the hallmark EMT gene signature and glycolysis (Fig. 2A and B; Supplementary Fig. S2A and S2B). Conversely, the HMLE/LacZ expression pattern displayed enrichment of genes associated with oxidative phosphorylation, consistent with the Warburg metabolic switch downstream of FOXQ1 and SNAI1 (Fig. 2C; Supplementary Fig. S2C).

We also observed that the 1,041 genes commonly upregulated in both SNAI1 and FOXQ1 cell models displayed positively correlative expression (*R* = 0.55, Spearman), supporting the potential for these genes to function as a network (Fig. 2D). RTKs have been historically successful pharmacologic targets in cancer treatment (46–48). Therefore, we sought to uncover the mechanistic link between RTKs and both the FOXQ1 and SNAI1 transcriptional axes. We examined 1,041 commonly upregulated gene subsets for the enrichment of gene signatures coexpressed with RTKs [ARCHS4 (49)]. Interestingly, the common gene set between FOXQ1/SNAI1 is highly enriched for a DDR2 expression signature, second only to PDGFRβ, a well-known EMT signaling RTK (refs. 41, 50–52; Fig. 2E). Collagen is a well-characterized ligand of the DDR family of RTKs. In line with this, the FOXQ1-SNAI1 upregulated gene set was also enriched for collagen metabolic processes and ECM remodeling (Fig. 2F). Together, these data suggest the DDR2 pathway and collagen metabolism could be critical overlapping functions downstream of FOXQ1 and SNAI1.

This finding prompted us to further explore the expression pattern of 48 RTKs regulated by either FOXQ1 or SNAI1 in HMLE cells using qRT-PCR. We found DDR2 to be the most dramatically upregulated RTK in both HMLE/FOXQ1 and HMLE/SNAI1 cells (Fig. 2G). *PDGFRβ*, *FGFR1* (fibroblast growth factor receptor 1), *ROR2* (receptor tyrosine kinase-like orphan receptor 2), *MuSK* (muscle-specific kinase), and *ROS* (ROS proto-oncogene) were also observed to be commonly upregulated between the FOXQ1 and SNAI1 EMT models (Fig. 2G). Western blot analysis further confirmed that these RTKs are shared effectors of FOXQ1 and SNAI1 (Fig. 2H).

### DDR2 is Preferentially Expressed in Basal-like Breast Cancer

**FIGURE 3:**
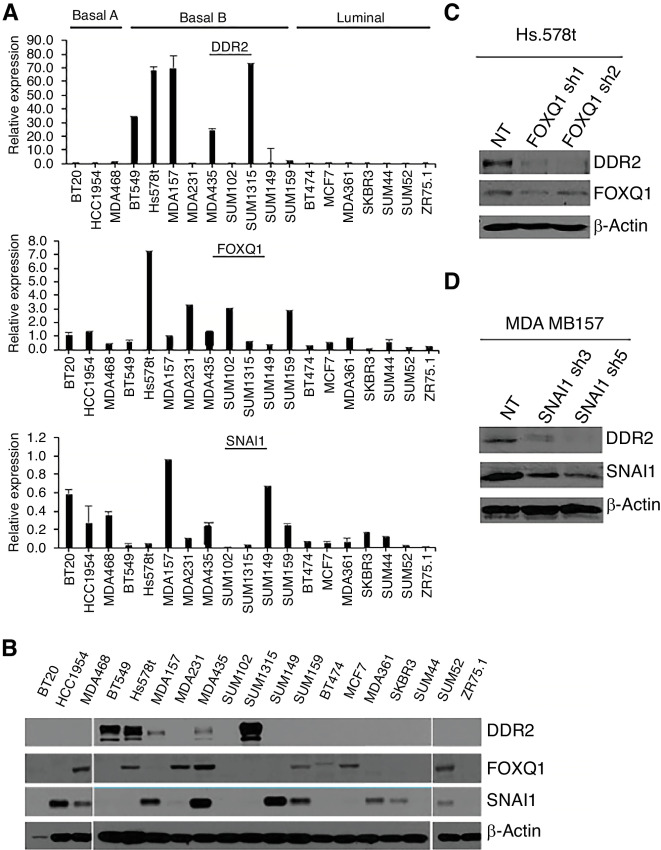
Investigation of DDR2 expression in breast cancer cell lines. **A,** DDR2 (top), FOXQ1 (middle), and SNAIL1 (bottom) mRNA expression was examined by qRT-PCR in a panel of human breast cancer cell lines. **B,** DDR2, FOXQ1, and SNAI1 protein expression were detected by Western blot analysis in a panel of human breast cancer cell lines. **C,** DDR2 protein was examined by Western blotting in Hs.578t cells with FOXQ1 knockdown or NT control. **D,** DDR2 protein was examined by Western blotting in MDA-MB 157 cells with SNAI1 knockdown and NT control.

We next examined the expression of the *DDR2* gene in a panel of breast cancer cell lines. *DDR2* showed preferential expression in the basal-b subtype of breast cancer cell lines. Specifically, *DDR2* was highly expressed in five basal-like breast cancer cell lines, including BT549, Hs. 578t, MDA-MB157, SUM 1315, and MDA-MB435 cells (Fig. 3A). BT549 and MDA1315 cell lines exhibited elevated expression of *DDR2* with undetectable *FOXQ1* and *SNAI1* expression. We further observed that *FOXQ1* and *SNAIL1* mRNA was highly expressed in two (Hs.578t and MDA-MB157) of the other three cell lines in a complementary pattern. Both TFs displayed overexpression in MDA MB435 cells, a cell line with questionable identity. The patterns of DDR2, FOXQ1, and SNAI1 expression were confirmed by western blot analysis in the same panel of cell lines (Fig. 3B). To further confirm the FOXQ1-DDR2 or SNAI1-DDR2 axis in cancer cells, we knocked down the *FOXQ1* gene in Hs.578t cells, and the *SNAIL1* gene in MDA MB157 cells since each of these cell lines displayed high expression of either *FOXQ1* or *SNAIL1* with comparatively low expression of the other. Upon FOXQ1 or SNAI1 knockdown, we observed concomitant decrease in DDR2 expression (Fig 3C and D).

Because DDR2 has been repeatedly found to drive metastasis in multiple cancer types, including breast cancer, we examined DDR2 expression in a set of mouse breast cancer cell lines that demonstrated differential metastatic capabilities *in vivo* (35). The highest expression of DDR2 was presented in the most metastatic cell line 4T1, as shown by qRT-PCR and Western blot analysis (Supplementary Fig. S3A and S3B).

We next utilized TCGA and METABRIC breast cancer datasets to explore the correlation of *DDR2* with other components of the shared *FOXQ1-SNAIL* gene set. A subset of the commonly upregulated *FOXQ1/SNAIL* RTKs displayed a significant positive correlation with *DDR2* expression, including *FGFR1*, *PDGFRA/PDGFRB*, and *KDR* (kinase insert domain receptor; Supplementary Fig. S3C and S3D). In addition, a subset of ECM components found to be downstream of FOXQ1/SNAI1 exhibited a significant positive correlation with DDR2, including multiple collagens, biglycan, and decorin (Supplementary Fig. S3E and S3F). Moreover, *DDR2* expression was significantly higher in TNBC than in hormone receptor-positive tumors (Supplementary Fig. S3G and S3H). In addition, analysis of gene expression data from TCGA revealed that *DDR2* expression is independently correlated with either *FOXQ1* or *SNAI1* expression (Supplementary Fig. S3I and S3J), and *DDR2* expression is highly associated with a 238-gene EMT signature (ref. 42; Supplementary Fig. S3K).

### DDR2 Knockdown has a Limited Effect on Cell Morphology and EMT in Human Mammary Epithelial Cells

**FIGURE 4:**
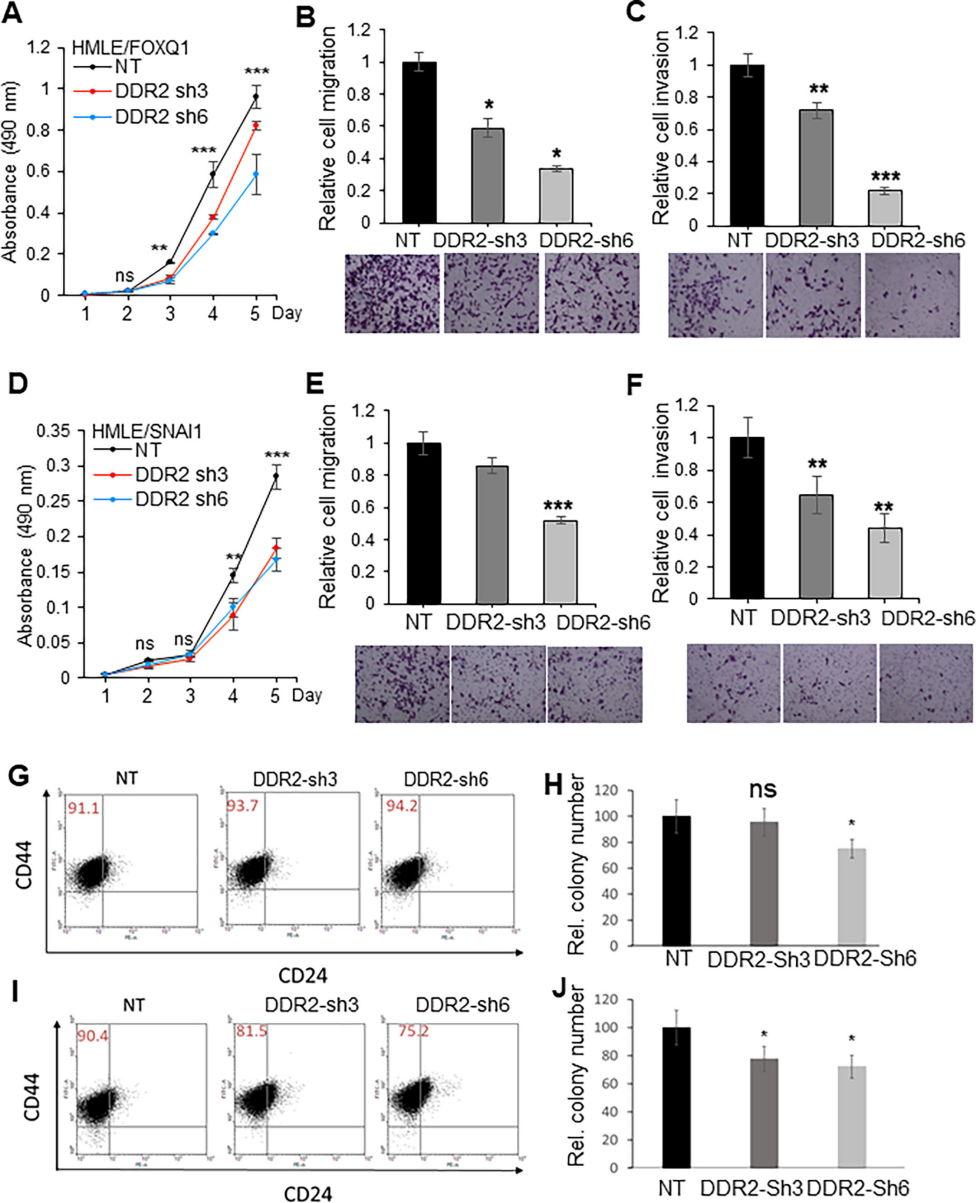
The effect of DDR2 on oncogenic properties and stem cell-like population in EMT cell model. **A,** The effect of DDR2 knockdown on HMLE/FOXQ1 cell proliferation. The effect of DDR2 knockdown on cell migration (**B**) and invasion (**C**) in HMLE/FOXQ1 cells. Bottom panels show representative pictures of migrated and invasive cells in **B** and **C**. **D,** The effect of DDR2 knockdown on HMLE/SNAI1 cell proliferation. The effect of DDR2 knockdown on cell migration (**E**) and invasion (**F**) in HMLE/SNAI1 cells. Bottom panels show representative pictures of migrated and invasive cells in **G** and **H**. For all panels, *, *P* < 0.05; **, *P* < 0.01; and ***, *P* < 0.001. **G,** Flow cytometry analysis of cell-surface markers, CD44 and CD24, in HMLE/FOXQ1 cells with DDR2 shRNA knockdown or NT control. **H,** Quantification of mammospheres formed by cells described in **A**. **I,** Flow cytometry analysis of cell-surface markers CD44 and CD24 in HMLE/SNAI1 cells with and without DDR2 knockdown. **J,***In vitro* quantification of mammospheres formed by cells described in **C**. For **B** and **D**, the data are reported as the number of mammospheres formed/1,000 seeded cells ± SEM, compared with control (two experiments performed in triplicate, *, *P* < 0.05).

To study the functional role of DDR2 in the EMT program, we performed DDR2 shRNA knockdown in both HMLE/FOXQ1 and HMLE/SNAI1 cell lines. Two clones (sh3 and sh6), that showed the most significant DDR2 knockdown in both EMT models, were selected for further functional studies (Supplementary Fig. S4A and S4B). Somewhat unexpectedly, we did not observe DDR2 knockdown significantly impacting the EMT phenotype of either HMLE/FOXQ1 or HMLE/SNAI1 cells. Western blot analysis displayed no significant alterations in the expression of epithelial cell markers, Occludin, and β-catenin, or mesenchymal markers, Fibronectin and N-cadherin, upon DDR2 knockdown in either HMLE/FOXQ1 or HMLE/SNAI1 cell models (Supplementary Fig. S4C and S4D). Furthermore, we found that knockdown of DDR2 did not lead to a marked change in the spindle-like, scattered distribution or morphology of either HMLE/FOXQ1 or HMLE/SNAI1 cells (Fig. 4E and F). Consistent with these results, we did not observe a change in vimentin expression or distribution using the same cell lines via immunofluorescence staining (Supplementary Fig. S4G and S4H).

### DDR2 Knockdown Strongly Influences Cell Motility in Human Mammary Epithelial Cells

To evaluate the effect of DDR2 on the biological characteristics of mammary epithelial cells, we performed a series of *in vitro* functional studies. First, we found that the knockdown of DDR2 in the HMLE/FOXQ1 EMT model modestly decreased cell proliferation (Fig. 4A). Consistent with this result, DDR2 knockdown resulted in considerably reduced HMLE/FOXQ1 cell migration (45% and 65% decrease in the sh3 and sh6 clones, respectively) and cell invasion (30% and 80% decrease in the sh3 and sh6 clones, respectively; Fig. 4B and C).

Similar results were also obtained in HMLE/SNAI1 cells. The knockdown of DDR2 in HMLE/SNAI1 cells resulted in a moderate decrease in cell proliferation (Fig. 4D). In addition, cell migration and invasion capacities were also significantly inhibited by DDR2 knockdown. Specifically, both sh3 and sh6 clones showed around a 50% decrease in cell migration compared with HMLE/SNAI1 NT cells (Fig. 4E). We observed a 40% and 60% decrease in cell invasion in HMLE/SNAI1 DDR2 sh3 and sh6 clones, respectively (Fig. 4F). To eliminate possible off-target effects of DDR2 knockdown, we performed DDR2 knockdown in HMLE/LacZ cells. We observed no apparent alterations in cell morphology, EMT marker expression, cell proliferation, or cell migration and invasion between HMLE/LacZ NT and DDR2 shRNA knockdown counterparts (Supplementary Fig. S5).

### DDR2 Knockdown has a Limited Effect on Stem Cell Abundance and Chemoresistance in Human Mammary Epithelial Cells

We next analyzed cells for the expression of stem cell markers CD44 and CD24 by flow cytometry. As expected, more than 90% of the HMLE/FOXQ1 NT cells have the CD44^+^/CD24^−^ expression pattern indicative of the EMT-acquired stem-like phenotype (ref. 45; Fig. 4G). Knockdown of DDR2 in the HMLE/FOXQ1 model did not decrease the CD44^+^/CD24^−^ population in the DDR2 sh3 and sh6 cells compared with the DDR2-NT model. Mammosphere formation assays were also performed to investigate the impact on acquired stemness and resulted in a minor 20% decrease in only the DDR2-sh6 cell model (Fig. 4H). DDR2 knockdown in the HMLE/SNAI1 cell model led to about a 10% and 15% decrease in the CD44^+^/CD24^−^ population in the DDR2 sh3 and sh6 clones, respectively (Fig. 4I). Similarly, DDR2 knockdown showed a comparable reduction in mammosphere formation in both sh3 and sh6 cell models, consistent with the observed alteration of stem cell population abundance (Fig. 4J).

To investigate whether DDR2 contributes to FOXQ1- or SNAI1-driven chemotherapy resistance, we tested for response to treatment with two conventional chemotherapeutic agents used as the standard of care in TNBC, Dox and Pac, in HMLE/FOXQ1 and HMLE/SNAI1 cell models. As measured by MTT assay, we did not observe a significant difference in cell viability to either Dox or Pac treatment in cells with DDR2 knockdown compared with respective NT control cells for either HMLE/FOXQ1 or HMLE/SNAI1 cell models (Supplementary Fig. S6A–S6D). These data suggest that DDR2 expression does not contribute to the resistance to chemotherapeutic agents in the mammary epithelial cell line.

### DDR2 Expression Altered Cell Motility in TNBC Cells without Impacting EMT

**FIGURE 5:**
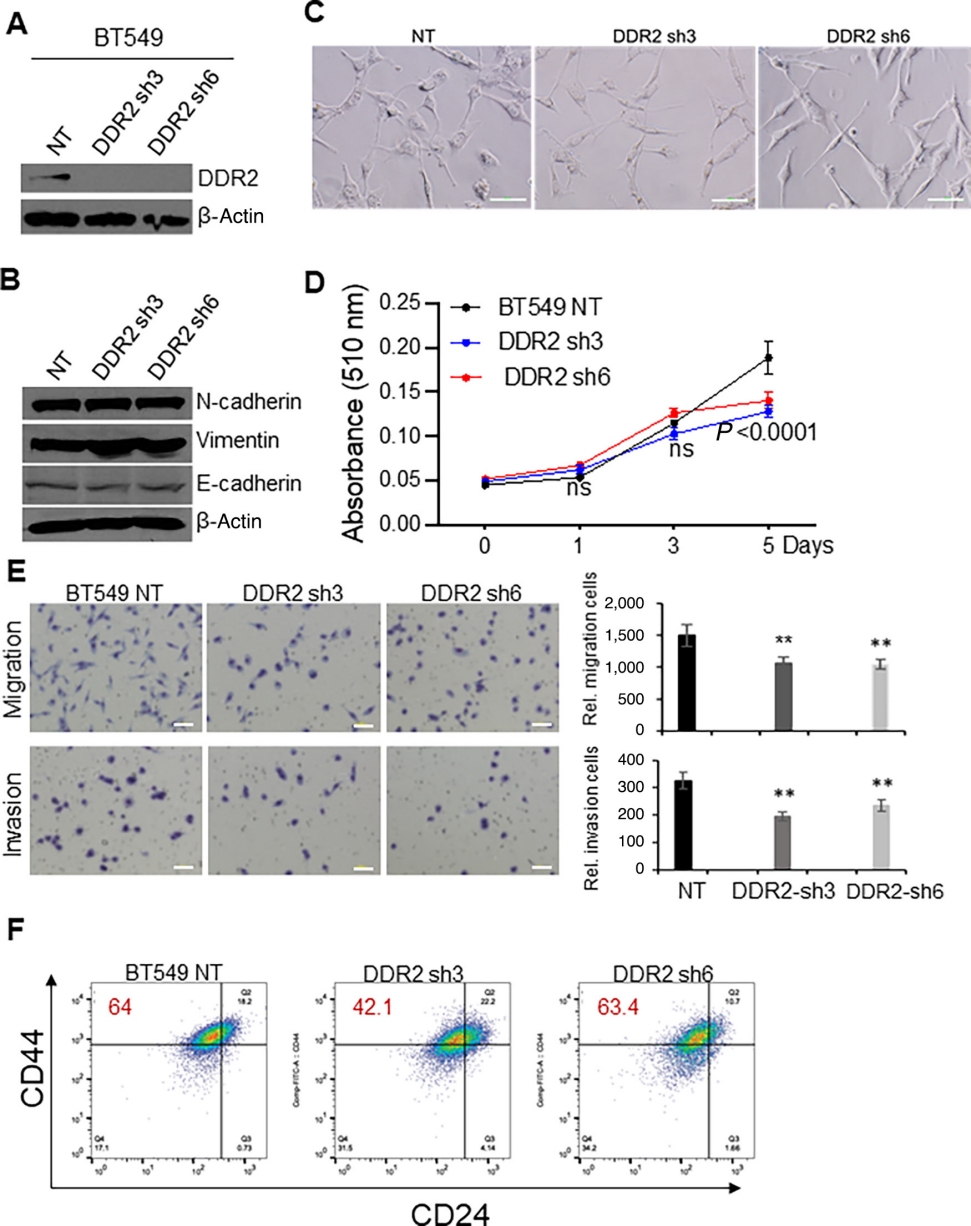
The effect of DDR2 knockdown on oncogenic properties and stem cell population in BT549 cells. **A,** Western blot analysis confirmed the knockdown of DDR2 in two clones derived from BT549 cells. **B,** Western blot analysis for mesenchymal markers VIM and N-cadherin and epithelial marker CDH1 was performed in the BT549 cells with or without DDR2 knockdown. **C,** Cell morphology of BT549 cells with or without DDR2 knockdown remains unchanged. Scale bar: 100 μm. **D,** Cell proliferation in the BT549 cells with or without DDR2 knockdown was measured by Sulforhodamine B assay. **E,** Cell migration and invasion assay was performed in the BT549 cells with or without DDR2 knockdown. A representative image for cell migration and invasion was shown in the left panels. The summary of the migration and invasion was shown in the right panels. Scale bar: 100 μm. **, *P* < 0.01. **F,** Flow cytometry analysis showed no marked changes in the CD44^+^/CD24^−^ population in BT549 cells with or without DDR2 knockdown.

Next, we sought to clarify whether the biological effects of DDR2 in TNBC cells mirror our observations from the human mammary epithelial cell. We generated DDR2 knockdown models in BT549 cells, which display minimal expression of FOXQ1 or SNAI1 (Fig. 5A). No significant change in the expression of EMT markers was observed in BT549 cells upon DDR2 knockdown (Fig. 5B). In line with this, no morphological change was observed in two DDR2 knockdown cell models relative to NT control (Fig. 5C). Moreover, we found that knockdown of DDR2 led to a marked decrease in cell proliferation (Fig. 5D), migration and invasion (Fig. 5E). However, DDR2 knockdown did not markedly alter the abundance of the CD44^+^/CD24^−^ population in BT549 cells compared with NT control (Fig. 5F).

**FIGURE 6:**
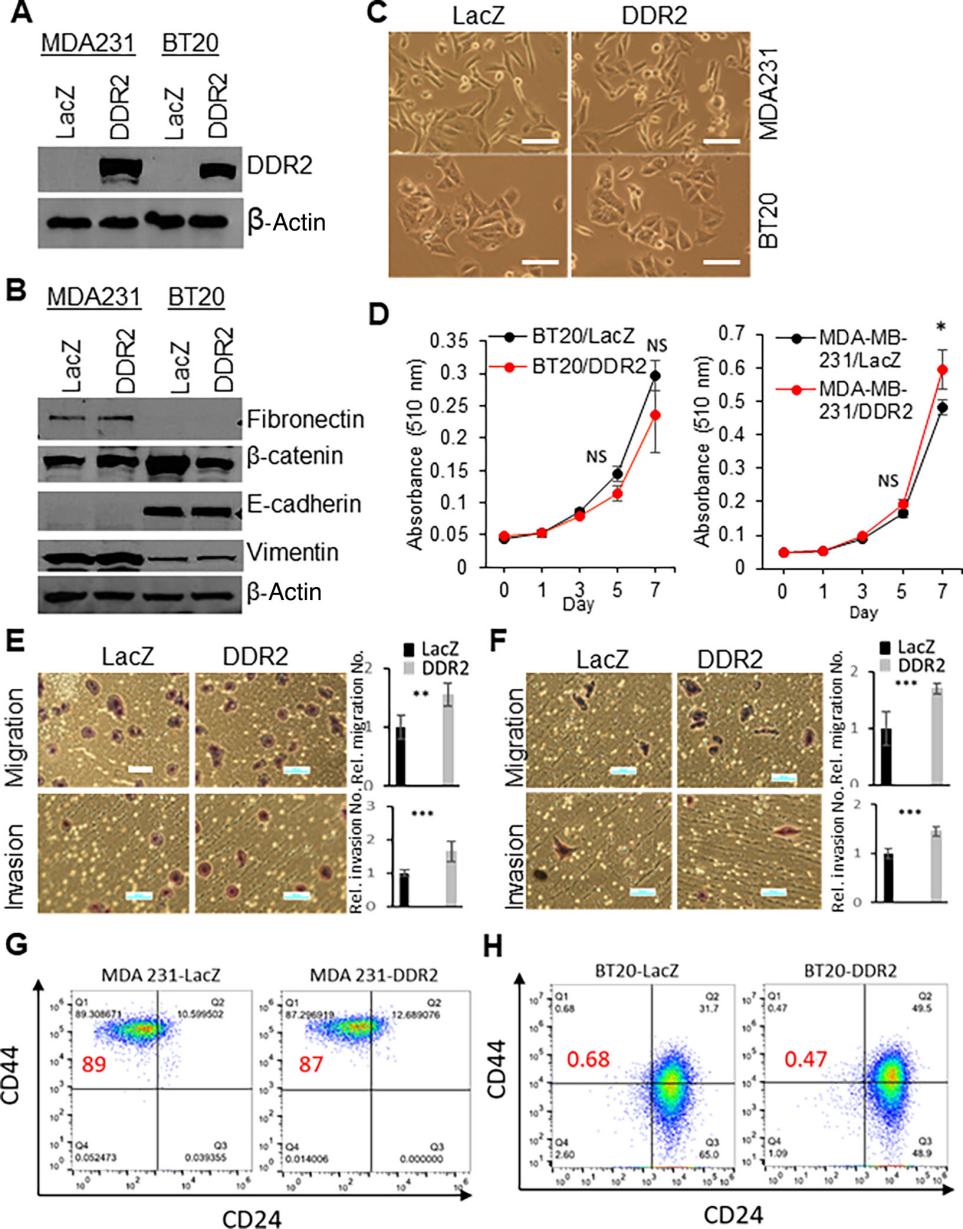
The effect of ectopic expression of DDR2 on oncogenic properties and stem cell population in MDA-MB 231 and BT20 cells. **A,** Western blot analysis confirmed the ectopic expression level of DDR2 in MDA231 and BT20 cells. **B,** Western blot analysis for mesenchymal markers Fibronectin and VIM and epithelial marker β-catenin and CDH1 was performed in MDA231 and BT20 cells with or without ectopic expression of DDR2. **C,** Cell morphology of MDA231 and BT20 cells with or without ectopic expression of DDR2 remains unchanged. Scale bar: 100 μm. **D,** Cell proliferation in MDA231 and BT20 cells with or without ectopic expression of DDR2 was measured by Sulforhodamine B assay. Cell migration and invasion assay were performed in the MDA231 (**E**) and BT20 (**F**) cells with or without ectopic expression of DDR2. A representative image for cell migration and invasion was shown in the left panels. The summary of the migration and invasion was demonstrated in the right panels. Scale bar: 100 μm. ***, *P* < 0.001. Flow cytometry analysis revealed no marked changes in the CD44^+^/CD24^−^ population in MDA231 (**G**) and BT20 (**H**) cells with or without ectopic expression of DDR2.

To validate the results obtained with the loss of function models, we ectopically expressed DDR2 in MDA-MB231 and BT20 cells (Fig. 6A). We found that ectopic expression of DDR2 did not lead to apparent EMT or morphologic change in either overexpression model (Fig. 6B and C). Moreover, we observed that DDR2 overexpression led to a minor increase in the cell proliferation of MDA-MB231 cells but had no significant effect in the BT20 cell model. (Fig. 6D). However, we did observe an increase in cell migration and invasion in both cell models upon DDR2 overexpression (Fig. 6E and F). No marked alteration in the abundance of the CD44^+^/CD24^−^ population was shown in either cell model with DDR2 overexpression compared with the LacZ vector control (Fig. 6G and H).

### Knockdown of DDR2 Reversed EMT- and Metastasis-related Metabolites

Because many of the genes downstream of both SNAI1-FOXQ1 and within the DDR2 coexpression signature are associated with metabolic functions, we hypothesized that DDR2 might contribute to the regulation of metabolism. In support of this, we observed that *DDR2* expression was robustly correlated with 42-gene mesenchymal metabolic signature (28) across TCGA breast cancer samples (*R* = 0.84, Spearman; Supplementary Fig. S7A).

**FIGURE 7:**
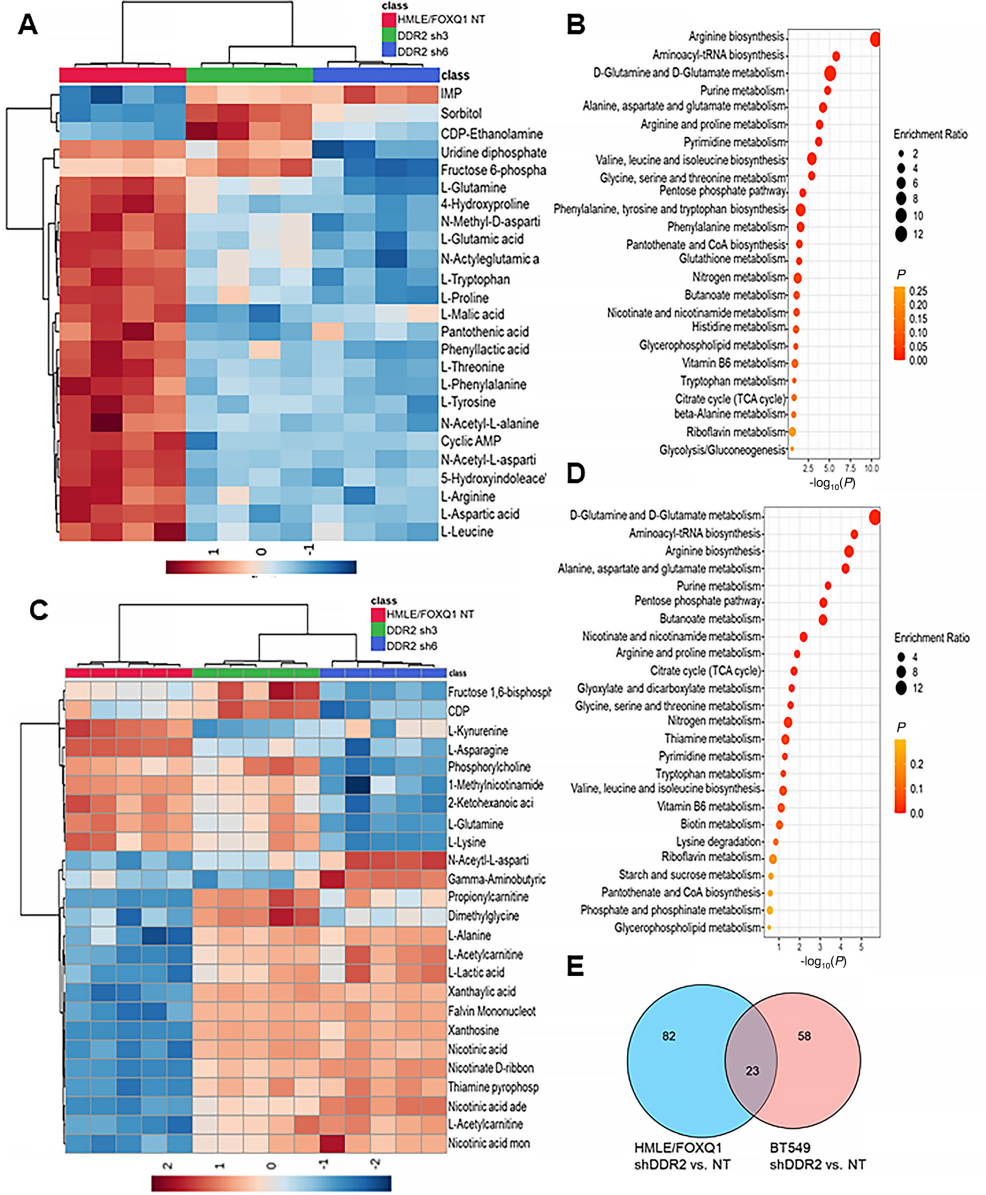
DDR2 contributes to metabolic alterations in EMT and TNBC cell models. **A,** Heatmap visualization of the top 25 differentially abundant metabolites between the HMLE/FOXQ1 NT control and shDDR2 knockdown cell models, significance determined by ANOVA. Samples and metabolites were subjects to unsupervised hierarchical clustering. Heatmap depicts the normalized metabolite concentrations across samples. **B,** Metabolite functional enrichment analysis of the 106 metabolites affected by DDR2 knockdown in HMLE/FOXQ1 cells using KEGG database functional annotation, top 25 enriched pathways are shown. **C,** Heatmap visualization of the top 25 differentially abundant metabolites between the BT549 NT control and shDDR2 knockdown cell models, significance determined by ANOVA. Samples and metabolites were subjects to unsupervised hierarchical clustering. Heatmap depicts the normalized metabolite concentrations across samples. **D,** Metabolite functional enrichment analysis of the 82 metabolites was significantly altered by DDR2 knockdown in BT549 cells using KEGG database functional annotation system; the top 25 enriched pathways are shown. **E,** Venn diagram of the overlap of metabolites that are altered upon DDR2 knockdown in HMLE/FOXQ1 and BT549 cells relative to the respective NT control cells.

To determine the metabolic pathways related to DDR2, we performed LC-MS/MS targeted metabolomic profiling (254 metabolites) of the HMLE/FOXQ1 EMT cell model with two stable DDR2 shRNA knockdown derivatives alongside NT control. The normalized intracellular metabolite concentrations and statistical analysis are presented as Extended data 2. Principal component analysis (PCA) confirmed that the two DDR2 knockdown cell lines have a distinct metabolic profile compared with HMLE/FOXQ1 NT control cells (Supplementary Fig. S7B). Metabolite concentrations were log-transformed and differentially regulated metabolites were determined by ANOVA. Globally, we identified 106 differentially regulated metabolites across the three groups (FDR = 0.10, Extended data 2). The top 25 most significantly altered metabolites were visualized by unsupervised hierarchical clustering (Fig. 7A, ANOVA).

The functions of 105 metabolites dysregulated by DDR2 reduction were subject to metabolite set enrichment analysis using the SMPDB and Kyoto Encyclopedia of Genes and Genomes (KEGG) annotation libraries. Only pathways with two or more differentially abundant metabolites were utilized for analysis. The results identified differential flux in amino acid pathways and the urea cycle, purine synthesis, and phosphatidylcholine/phosphatidyenolamine biosynthesis (MetaboAnalyst; Fig. 7B; Supplementary Fig. S7C). Changes in metabolic pathways were also analyzed by metabolic pathway hypergeometric test using KEGG annotation [MetaboAnalyst (53)]. These results identified dysregulation of amino acid metabolism, including arginine biosynthesis, alanine/aspartate/glutamate metabolism, and phenylalanine/tyrosine/tryptophan biosynthesis (Supplementary Fig. S7D). We also observed dysregulation of metabolites enriched for Warburg metabolism, which is consistent with our previous findings of a Warburg gene signature downstream of FOXQ1 (Supplementary Fig. S7D).

We further sought to analyze the effects of DDR2 on cellular metabolism in TNBC cells by performing metabolomic profiling of BT549 cells with shRNA knockdown of DDR2 (DDR2 sh3, DDR2 sh6) alongside NT control. PCA confirmed that the metabolic profiles of the DDR2 knockdown samples are distinct from those of the NT control samples in BT549 cells (Supplementary Fig. S7E). Overall, we identified 82 metabolites that are significantly altered upon DDR2 knockdown in BT549 cells (ANOVA, FDR = 0.1, Extended data 3). The top 25 metabolites are shown in Fig. 7C. We interrogated the metabolic pathways that were most significantly altered by DDR2 knockdown in BT549 cells by enrichment analysis against the KEGG and SMPDB annotation databases (Fig. 7D; Supplementary Fig. S7F). The most significantly altered pathways included d-glutamine and d-glutamate metabolism, aminoacyl-tRNA biosynthesis and arginine biosynthesis according to KEGG annotation (Fig. 7D). Consistent with these findings, enrichment using SMPDB annotation system identified Glutamine metabolism as the most significantly altered pathway impacted by DDR2 silencing (Supplementary Fig. S7F). We applied another approach to functional pathway interrogation using hypergeometric testing, which confirmed that several amino acid pathways were impacted including glutamine and glutamate metabolic processes (Supplementary Fig. S7G).

**FIGURE 8:**
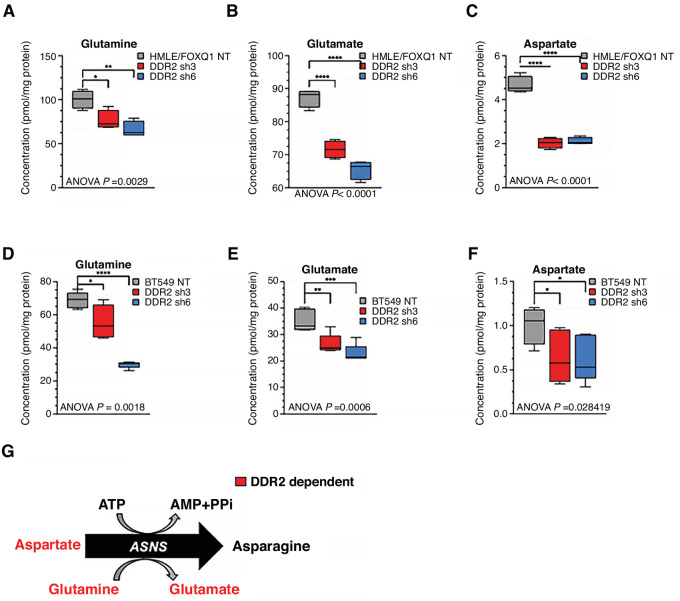
Metabolites in asparagine synthesis were repressed upon DDR2 knockdown. **A–G,** Metabolite concentrations of the glutamine (**A**), glutamine (**B**), and aspartate (**C**) in HMLE/FOXQ1 cell models were quantified by metabolomics. Metabolite concentrations of the glutamine (**D**), glutamine (**E**), and aspartate (**F**) in BT549 cell models were quantified by metabolomics **G,** Overview of aspartate/asparagine metabolic flux. DDR2-dependent metabolites were highlighted in red. For all panels, *, *P* < 0.05; **, *P* < 0.01; and ***, *P* < 0.001.

Finally, we sought to analyze the DDR2-dependent metabolic processes in both the FOXQ1-driven EMT and BT549 TNBC cell models. A comparison of the significantly altered metabolites in both cell models identified 50 metabolites that were altered in both model systems upon DDR2 knockdown (Fig. 7E). However, closer examination revealed the directional effects of DDR2 knockdown on metabolite abundance in the HMLE/FOXQ1 and BT549 cell lines were only conserved for 23 of these metabolites (19 downregulated and four upregulated; Fig. 7E; Supplementary Table S1). Among the shared metabolic alterations, we observed a significant decrease in glutamine, glutamate, and aspartate in both HMLE/FOXQ1 and BT549 cells upon DDR2 knockdown (Fig. 8A–F), suggesting a critical role of DDR2 signaling in the asparagine synthesis pathway (Fig. 8G). In addition, both HMLE/FOXQ1 and BT549 cell models displayed an increase in the abundance of glyceraldehyde-3-phosphate, a shared metabolite within the glycolytic and pentose phosphate pathways (Supplementary Fig. S8A). Moreover, DDR2 knockdown was associated with a decrease in threonine, lysine, isoleucine, and valine in both HMLE/FOXQ1 and BT549 cells (Supplementary Fig. S8A).

We also observed that DDR2 knockdown had distinct effects on cell metabolism in HMLE/FOXQ1 and BT549 cells. For example, while pathway analysis identified that DDR2 knockdown was associated with an alteration in the TCA cycle in both models, the metabolites impacted in this pathway were model specific. We found that knockdown of DDR2 in HMLE/FOXQ1 cells led to a decrease in fumarate, malate, and 2-oxoglutarate (Supplementary Fig. S8B–S8D). Meanwhile, BT549 DDR2 knockdown models displayed a succinate reduction and increased 2-oxoglutarate acid and 2-phosphoglycerate compared with BT549 NT control samples (Supplementary Fig. S8E–S8G). Knockdown of DDR2 resulted in a more dramatic impact on glycolysis in HMLE/FOXQ1 cells, as observed by a decrease in the abundance of fructose-6-phosphate, fructose-1,6-bisphosphate, and 3-phosphoglycerate (Supplementary Fig. S8A). Conversely, DDR2 knockdown was associated with increased 3-phosphoglycerate in BT549 cells. In addition, HMLE/FOXQ1 shDDR2 cells displayed a decrease in the abundance of alanine, leucine, tyrosine, phenylalanine, histidine, and proline (Supplementary Fig. S8A). Conversely, BT549 cells displayed an increase in alanine abundance and a unique decrease in serine abundance.

## Discussion

The primary goal of this study is to decipher the hierarchy of EMT-TFs in mammary epithelial cells and identify common EMT effectors. To this end, we have several findings in this current study that other research groups have not reported. First, we found that within the EMT-TF network, FOXQ1 and SNAI1 act upstream to induce the expression of core EMT-TFs including ZEB1/2, TWIST1, and FOXC2. We also reported that FOXQ1 and SNAI1 acted as parallel transcriptional axes with a shared capacity to induce EMT without reciprocally regulating the expression of one another. In line with this discovery, the transcription profiles of FOXQ1 and SNAI1 were functionally redundant in regulating a common set of EMT gene targets, along with distinct and unique regulatory profiles. Because FOXQ1 and SNAI1 were independently capable of inducing EMT, we reasoned that common downstream effectors could be critical nodes within the EMT network and potential therapeutic targets. We identified a common set of RTKs regulated by FOXQ1 and SNAI1 in mammary and TNBC cells, including DDR2. Further study is needed to validate this regulatory axis in tumor samples and to characterize the redundant and distinct biological functions of FOXQ1 and SNAI1 in cancer and normal physiology.

Second, we found that DDR2 is highly upregulated in FOXQ1- and SNAI1-driven EMT models and displays concomitant coexpression with either FOXQ1 or SNAI1 in TNBC cell lines. DDR2 expression was also independently correlated with FOXQ1 and SNAI1 across TCGA breast cancer data. Previous studies have shown that DDR2 promotes breast cancer metastasis through stabilizing SNAI1 (16, 54, 55). Therefore, our results, in conjunction with other published data, suggest a SNAI1/DDR2 mutual regulation loop that facilitates the sustained activation of the DDR2 signaling pathway in breast tumor metastatic progression. Interestingly, TWIST1 has been reported to regulate DDR2 expression in ovarian cancer (17) and was found to be a shared effector of FOXQ1 and SNAI1 in this study. Future work is needed to uncover the distinct or shared mechanisms of epigenetic regulation of DDR2 in the various EMT contexts.

Third, although DDR2 has been implicated in EMT in multiple cancer types (13, 14, 18, 54), we observed that alteration of DDR2 expression by ectopic expression or knockdown did not alter the EMT phenotype in the HMLE TF-driven models or multiple TNBC cell models. Instead, we observed DDR2 most prominently impacted cell motility and displayed variable effects on cell proliferation and stem-like phenotype in a cell line–specific manner. These results are consistent with several other studies that show conflicting evidence for the role of DDR2 in the regulation of EMT, proliferation, and cell adhesion in cancer (16, 56). These contradictions have been observed for metastasis as well. While many studies have found DDR2 playing a prometastatic function, DDR2-null mice were found to have a 3-fold increase in colon cancer metastasis (57). Therefore, it is clear that the cellular contexts and the cues from the microenvironment are crucial for dictating DDR2 function. The contexts by which DDR2 does have a feedback role in the regulation of EMT need to be empirically determined.

The identification of common metabolites and numerous model-specific metabolites highlights the biological connection and diversity between EMT and TNBC cell models. For example, we found that DDR2 knockdown significantly impacted glycolysis in HMLE/FOXQ1 but not in BT549 cells, as shown by a reduction in the abundance of five of the 11 critical glycolytic metabolites. This may be explained by the fact that HMLE cells are immortalized but have not undergone an oncogenic transformation. Because the glycolytic pathway is critical for cancer cell proliferation and maintenance, BT549 cells may have acquired redundant regulatory mechanisms to maintain glycolytic flux without DDR2. In contrast, HMLE/FOXQ1 cells may be more reliant on DDR2 downstream signaling for the regulation of glycolysis in the absence of additional oncogenic alterations. Importantly, we found a marked decrease in different forms of aspartic acid (aspartate) along with glutamine and glutamate among 23 common altered metabolites, suggesting a deficient asparagine synthesis in DDR2 knockdown cells. Asparagine is an essential amino acid for protein synthesis needed to adapt to the relatively low levels of extracellular glutamine in cancer cells (58–61). Suppression of the bioavailability of asparagine through dietary restriction or L-asparaginase, which catalyzes the hydrolysis of asparagine to aspartic acid and ammonia, leads to suppression of breast cancer metastasis (62). Consistent with these findings, our results in the EMT and TNBC cell models suggest DDR2 may exert regulatory effects on the asparagine and glutamine synthesis, thereby promoting adaptation to cell stress and protein synthesis. Therefore, targeting asparagine synthesis may combat the protumorigenic functions of DDR2.

However, the mechanism of how DDR2 regulates global metabolism remains unknown. In a preliminary analysis of the correlation of DDR2 expression and 42-mesenchymal metabolism enzymes across TCGA breast cancer samples, we found a robust positive correlation (*R* = 0.84, Spearman; Fig. 7A). This suggests that the DDR2 signaling pathway may modulate the metabolic status of cancer cells through regulating the expression of certain metabolism enzymes or through signaling changes that impact enzymatic activity through posttranslational modification. Future work may focus on tracing the metabolic flux through isotope labels to understand the critical enzymes regulated downstream of DDR2. These insights could lay the foundation for exploring a specific understanding of the cellular contexts that dictate the phenotypic output of DDR2 regulation and for uncovering critical metabolic targets that mediate the protumorigenic effects of DDR2 in cancer cells.

## Supplementary Material

Supplementary Figures 1-8, Table 1 Supplementary Figure 1. Gene ontology molecular function and PANTHER pathway enrichment analysis for genes uniquely dyregulated in HMLE/FOXQ1 and HMLE/SNAI1 cells. Supplementary Figure 2. Gene set enrichment analysis (GSEA) based on 2201 genes commonly dyregulated in HMLE/FOXQ1 and HMLE/SNAL1 cells. Supplementary Figure 3. DDR2 expression in breast cancer. Supplementary Figure 4. DDR2 has minimal effect on EMT. Supplementary Figure 5. The effect of DDR2 on cell proliferation, EMT, cell migration, and invasion in HMLE/LacZ control cells. Supplementary Figure 6. The effect of DDR2 on stemness properties in EMT cell models. Supplementary Figure 7. The effect of DDR2 knockdown on cellular metabolism. Supplementary Figure 8. Model-specific metabolites changes in HMLE/FOXQ1 cells (EMT) and BT549 cells (TNBC) within the TCA. Supplementary Table 1. Twenty-three metabolites commonly dysregulated by DDR2 knockdown in HMLE/FOXQ1 and BT549 cells.

Supplementary Primers and shRNA Information Information of PCR primers for all gene tested and information for shRNA sequences for targeting several genes.

Extended Dataset 1 Common deregulated genes between HMLE_FOXQ1 and HMLE_SNAIL1 cell lines

Extended Dataset 2 Metabolomics analysis of comparison between HMLE_FOXQ1 and HMLE_FOXQ1_shDDR2 cell models

Extended Dataset 3 Metabolomics analysis of comparison between BT549_NT and BT549_shDDR2 cell models
