# Supplementary material for: DDR2 Coordinates EMT and Metabolic Reprogramming as a Shared Effector of FOXQ1 and SNAI1
Source: Cancer Res Commun. 2022 Nov 9;2(11):1388–403. doi: 10.1158/2767-9764.CRC-22-0013 (PMC9881645; doi:10.1158/2767-9764.CRC-22-0013)
Supplement: Supplementary Figures 1-8, Table 1 — Supplementary Figure 1. Gene ontology molecular function and PANTHER pathway enrichment analysis for genes uniquely dyregulated in HMLE/FOXQ1 and HMLE/SNAI1 cells. Supplementary Figure 2. Gene set enrichment analysis (GSEA) based on 2201 genes commonly dyregulated in HMLE/FOXQ1 and HMLE/SNAL1 cells. Supplementary Figure 3. DDR2 expression in breast cancer. Supplementary Figure 4. DDR2 has minimal effect on EMT. Supplementary Figure 5. The effect of DDR2 on cell proliferation, EMT, cell migration, and invasion in HMLE/LacZ control cells. Supplementary Figure 6. The effect of DDR2 on stemness properties in EMT cell models. Supplementary Figure 7. The effect of DDR2 knockdown on cellular metabolism. Supplementary Figure 8. Model-specific metabolites changes in HMLE/FOXQ1 cells (EMT) and BT549 cells (TNBC) within the TCA. Supplementary Table 1. Twenty-three metabolites commonly dysregulated by DDR2 knockdown in HMLE/FOXQ1 and BT549 cells. [file crc-22-0013-s01.pdf]

DDR2 coordinates EMT and metabolic reprogramming as a shared effector of FOXQ1 and SNAI1

Allison V. Mitchell [*], Jason Wu [*], Fanyan Meng [*], Lun Dong, C James Block, Won-min Song, Bin Zhang, Jing Li, Guojun Wu [#]

1: Supplementary Figures and Table

2: Supplementary Primers and shRNA information

Supplementary Figures and Table

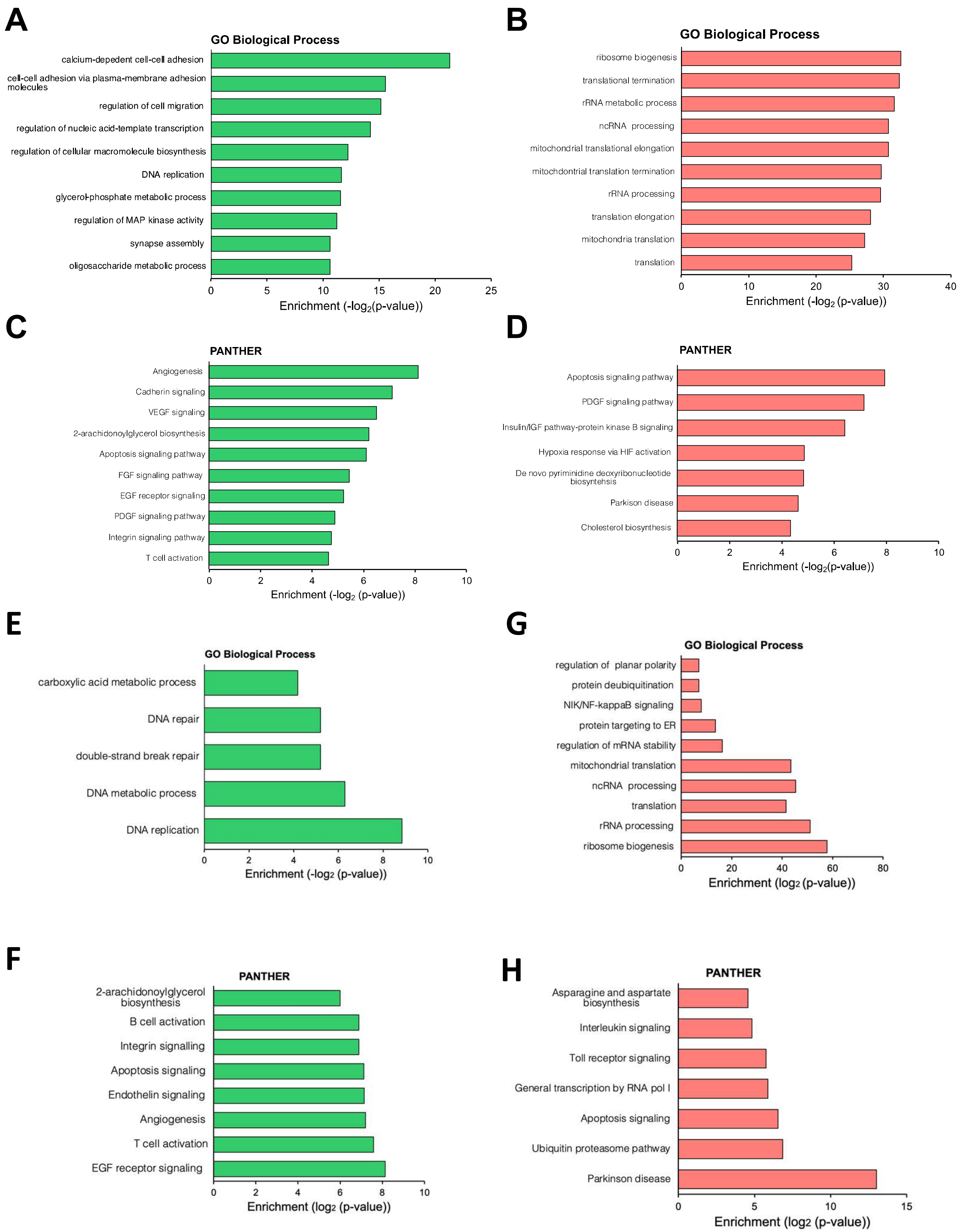

Supplementary Figure 1

Supplementary Figure 1. Gene ontology molecular function and PANTHER pathway enrichment analysis for genes uniquely dyregulated in HMLE/FOXQ1 and HMLE/SNAI1 cells. A-B. Hypergeometric tests of the genes that are uniquely upregulated in HMLE/SNAI1 (A) or HMLE/FOXQ1 (B) cells for terms in the Gene Ontology molecular function. **C-D.** PANTHER pathway enrichment analysis for the HMLE/SNAI1 (C) or HMLE/FOXQ1 (D) uniquely upregulated gene targets. **E.** Hypergeometric tests of the genes that are uniquely downregulated in HMLE/SNAI1 cells for terms in the Gene Ontology molecular function. **F.** PANTHER pathway enrichment analysis for the HMLE/SNAI1 cells uniquely downregulated gene targets. **G.** Hypergeometric tests of the genes that are uniquely downregulated in HMLE/FOXQ1 cells for terms in the Gene Ontology molecular function. **H.** PANTHER pathway enrichment analysis for the HMLE/FOXQ1 cells uniquely downregulated gene targets.

A

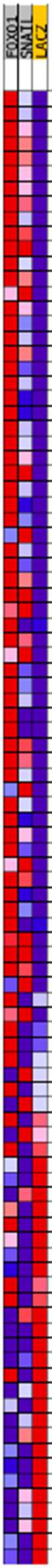

SampleName		
RGS4	RGS4	regulator of G protein signaling 4 [Source:HGNC Symbol;Acc:HGNC:10000]
COL3A1	COL3A1	collagen type III alpha 1 chain [Source:HGNC Symbol;Acc:HGNC:2201]
PRRX1	PRRX1	paired related homeobox 1 [Source:HGNC Symbol;Acc:HGNC:9142]
DCN	DCN	decorin [Source:HGNC Symbol;Acc:HGNC:2705]
ABI3BP	ABI3BP	ABI family member 3 binding protein [Source:HGNC Symbol;Acc:HGNC:17265]
FBLN5	FBLN5	fibulin 5 [Source:HGNC Symbol;Acc:HGNC:3602]
COL1A2	COL1A2	collagen type I alpha 2 chain [Source:HGNC Symbol;Acc:HGNC:2198]
BGN	BGN	biglycan [Source:HGNC Symbol;Acc:HGNC:1044]
COL1A1	COL1A1	collagen type I alpha 1 chain [Source:HGNC Symbol;Acc:HGNC:2197]
FBN1	FBN1	fibrillin 1 [Source:HGNC Symbol;Acc:HGNC:3603]
ANPEP	ANPEP	"alanyl aminopeptidase, membrane [Source:HGNC Symbol;Acc:HGNC:500]"
SLIT2	SLIT2	slit guidance ligand 2 [Source:HGNC Symbol;Acc:HGNC:11086]
WIPF1	WIPF1	WAS/WASL interacting protein family member 1 [Source:HGNC Symbol;Acc:HGNC:12736]
PTX3	PTX3	pentraxin 3 [Source:HGNC Symbol;Acc:HGNC:9692]
PDGFRB	PDGFRB	platelet derived growth factor receptor beta [Source:HGNC Symbol;Acc:HGNC:8804]
COL6A2	COL6A2	collagen type VI alpha 2 chain [Source:HGNC Symbol;Acc:HGNC:2212]
ITGB3	ITGB3	integrin subunit beta 3 [Source:HGNC Symbol;Acc:HGNC:6156]
POSTN	POSTN	periostin [Source:HGNC Symbol;Acc:HGNC:16953]
WNT5A	WNT5A	Wnt family member 5A [Source:HGNC Symbol;Acc:HGNC:12784]
COL5A2	COL5A2	collagen type V alpha 2 chain [Source:HGNC Symbol;Acc:HGNC:2210]
COL6A3	COL6A3	collagen type VI alpha 3 chain [Source:HGNC Symbol;Acc:HGNC:2213]
FOXC2	FOXC2	forkhead box C2 [Source:HGNC Symbol;Acc:HGNC:3801]
GREM1	GREM1	"gremlin 1, DAN family BMP antagonist [Source:HGNC Symbol;Acc:HGNC:2001]"
MGP	MGP	matrix Gla protein [Source:HGNC Symbol;Acc:HGNC:7060]
MMP2	MMP2	matrix metallopeptidase 2 [Source:HGNC Symbol;Acc:HGNC:7166]
TGFBR3	TGFBR3	transforming growth factor beta receptor 3 [Source:HGNC Symbol;Acc:HGNC:11774]
IGFBP4	IGFBP4	insulin like growth factor binding protein 4 [Source:HGNC Symbol;Acc:HGNC:5473]
EFEMP2	EFEMP2	EGF containing fibulin extracellular matrix protein 2 [Source:HGNC Symbol;Acc:HGNC:3219]
MYL9	MYL9	myosin light chain 9 [Source:HGNC Symbol;Acc:HGNC:15754]
PCOLCE	PCOLCE	procollagen C-endopeptidase enhancer [Source:HGNC Symbol;Acc:HGNC:8738]
FBN2	FBN2	fibrillin 2 [Source:HGNC Symbol;Acc:HGNC:3604]
LAMA1	LAMA1	laminin subunit alpha 1 [Source:HGNC Symbol;Acc:HGNC:6481]
THY1	THY1	Thy-1 cell surface antigen [Source:HGNC Symbol;Acc:HGNC:11801]
LRP1	LRP1	LDL receptor related protein 1 [Source:HGNC Symbol;Acc:HGNC:6692]
LOX	LOX	lysyl oxidase [Source:HGNC Symbol;Acc:HGNC:6664]
ADAM12	ADAM12	ADAM metallopeptidase domain 12 [Source:HGNC Symbol;Acc:HGNC:190]
GPX7	GPX7	glutathione peroxidase 7 [Source:HGNC Symbol;Acc:HGNC:4559]
OXTR	OXTR	oxytocin receptor [Source:HGNC Symbol;Acc:HGNC:8529]
VIM	VIM	vimentin [Source:HGNC Symbol;Acc:HGNC:12692]
BDNF	BDNF	brain derived neurotrophic factor [Source:HGNC Symbol;Acc:HGNC:1033]
FBLN1	FBLN1	fibulin 1 [Source:HGNC Symbol;Acc:HGNC:3600]
HTRA1	HTRA1	HtrA serine peptidase 1 [Source:HGNC Symbol;Acc:HGNC:9476]
SCG2	SCG2	secretogranin II [Source:HGNC Symbol;Acc:HGNC:10575]
TAGLN	TAGLN	transgelin [Source:HGNC Symbol;Acc:HGNC:11553]
ACTA2	ACTA2	"actin alpha 2, smooth muscle [Source:HGNC Symbol;Acc:HGNC:130]"
PMP22	PMP22	peripheral myelin protein 22 [Source:HGNC Symbol;Acc:HGNC:9118]
ID2	ID2	inhibitor of DNA binding 2 [Source:HGNC Symbol;Acc:HGNC:5361]
GEM	GEM	GTP binding protein overexpressed in skeletal muscle [Source:HGNC Symbol;Acc:HGNC:4234]
CDH2	CDH2	cadherin 2 [Source:HGNC Symbol;Acc:HGNC:1759]
MYLK	MYLK	myosin light chain kinase [Source:HGNC Symbol;Acc:HGNC:7590]
SPARC	SPARC	secreted protein acidic and cysteine rich [Source:HGNC Symbol;Acc:HGNC:11219]
LOXL2	LOXL2	lysyl oxidase like 2 [Source:HGNC Symbol;Acc:HGNC:6666]
FERMT2	FERMT2	fermitin family member 2 [Source:HGNC Symbol;Acc:HGNC:15767]
LOXL1	LOXL1	lysyl oxidase like 1 [Source:HGNC Symbol;Acc:HGNC:6665]
SERPINE1	SERPINE1	serpin family E member 1 [Source:HGNC Symbol;Acc:HGNC:8583]
NNMT	NNMT	nicotinamide N-methyltransferase [Source:HGNC Symbol;Acc:HGNC:7861]
FSTL1	FSTL1	follistatin like 1 [Source:HGNC Symbol;Acc:HGNC:3972]
DAB2	DAB2	DAB adaptor protein 2 [Source:HGNC Symbol;Acc:HGNC:2662]
RHOB	RHOB	ras homolog family member B [Source:HGNC Symbol;Acc:HGNC:668]
PCOLCE2	PCOLCE2	procollagen C-endopeptidase enhancer 2 [Source:HGNC Symbol;Acc:HGNC:8739]
BASP1	BASP1	brain abundant membrane attached signal protein 1 [Source:HGNC Symbol;Acc:HGNC:957]
PLOD2	PLOD2	"procollagen-lysine,2-oxoglutarate 5-dioxygenase 2 [Source:HGNC Symbol;Acc:HGNC:9082]"
CD59	CD59	CD59 molecule (CD59 blood group) [Source:HGNC Symbol;Acc:HGNC:1689]
ENO2	ENO2	enolase 2 [Source:HGNC Symbol;Acc:HGNC:3353]
LGALS1	LGALS1	galectin 1 [Source:HGNC Symbol;Acc:HGNC:6561]
TPM2	TPM2	tropomyosin 2 [Source:HGNC Symbol;Acc:HGNC:12011]
CALD1	CALD1	caldesmon 1 [Source:HGNC Symbol;Acc:HGNC:1441]
ITGA5	ITGA5	integrin subunit alpha 5 [Source:HGNC Symbol;Acc:HGNC:6141]
TNC	TNC	tenascin C [Source:HGNC Symbol;Acc:HGNC:5318]
DST	DST	dystonin [Source:HGNC Symbol;Acc:HGNC:1090]
GLIPR1	GLIPR1	GLI pathogenesis related 1 [Source:HGNC Symbol;Acc:HGNC:17001]
TPM1	TPM1	tropomyosin 1 [Source:HGNC Symbol;Acc:HGNC:12010]
ECM1	ECM1	extracellular matrix protein 1 [Source:HGNC Symbol;Acc:HGNC:3153]
SGCB	SGCB	sarcoglycan beta [Source:HGNC Symbol;Acc:HGNC:10806]
TGFB1	TGFB1	transforming growth factor beta 1 [Source:HGNC Symbol;Acc:HGNC:11766]
LAMC1	LAMC1	laminin subunit gamma 1 [Source:HGNC Symbol;Acc:HGNC:6492]
CALU	CALU	calumenin [Source:HGNC Symbol;Acc:HGNC:1458]
GADD45B	GADD45B	growth arrest and DNA damage inducible beta [Source:HGNC Symbol;Acc:HGNC:4096]
NOTCH2	NOTCH2	notch receptor 2 [Source:HGNC Symbol;Acc:HGNC:7882]
TGM2	TGM2	transglutaminase 2 [Source:HGNC Symbol;Acc:HGNC:11778]
COL7A1	COL7A1	collagen type VII alpha 1 chain [Source:HGNC Symbol;Acc:HGNC:2214]
GADD45A	GADD45A	growth arrest and DNA damage inducible alpha [Source:HGNC Symbol;Acc:HGNC:4095]
SNAI2	SNAI2	snail family transcriptional repressor 2 [Source:HGNC Symbol;Acc:HGNC:11094]
ITGA2	ITGA2	integrin subunit alpha 2 [Source:HGNC Symbol;Acc:HGNC:6137]
TNFAIP3	TNFAIP3	TNF alpha induced protein 3 [Source:HGNC Symbol;Acc:HGNC:11896]
CAP2	CAP2	cyclase associated actin cytoskeleton regulatory protein 2 [Source:HGNC Symbol;Acc:HGNC:20039]
VEGFA	VEGFA	vascular endothelial growth factor A [Source:HGNC Symbol;Acc:HGNC:12680]
TGFBI	TGFBI	transforming growth factor beta induced [Source:HGNC Symbol;Acc:HGNC:11771]
IL32	IL32	interleukin 32 [Source:HGNC Symbol;Acc:HGNC:16830]
CXCL1	CXCL1	C-X-C motif chemokine ligand 1 [Source:HGNC Symbol;Acc:HGNC:4602]
PMEPA1	PMEPA1	"prostate transmembrane protein, androgen induced 1 [Source:HGNC Symbol;Acc:HGNC:14107]"
SFRP1	SFRP1	secreted frizzled related protein 1 [Source:HGNC Symbol;Acc:HGNC:10776]
PTHLH	PTHLH	parathyroid hormone like hormone [Source:HGNC Symbol;Acc:HGNC:9607]
LAMA3	LAMA3	laminin subunit alpha 3 [Source:HGNC Symbol;Acc:HGNC:6483]
INHBA	INHBA	inhibin subunit beta A [Source:HGNC Symbol;Acc:HGNC:6066]
AREG	AREG	amphiregulin [Source:HGNC Symbol;Acc:HGNC:651]
MFAP5	MFAP5	microfibril associated protein 5 [Source:HGNC Symbol;Acc:HGNC:29673]
LAMC2	LAMC2	laminin subunit gamma 2 [Source:HGNC Symbol;Acc:HGNC:6493]

B

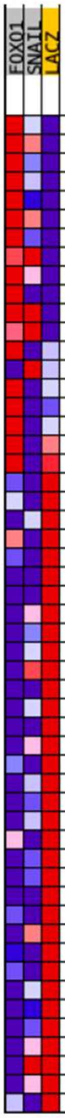

SampleName		
SDC2	SDC2	syndecan 2 [Source:HGNC Symbol;Acc:HGNC:10659]
DCN	DCN	decorin [Source:HGNC Symbol;Acc:HGNC:2705]
CHST2	CHST2	carbohydrate sulfotransferase 2 [Source:HGNC Symbol;Acc:HGNC:1970]
LHX9	LHX9	LIM homeobox 9 [Source:HGNC Symbol;Acc:HGNC:14222]
SDC3	SDC3	syndecan 3 [Source:HGNC Symbol;Acc:HGNC:10660]
STC1	STC1	stanniocalcin 1 [Source:HGNC Symbol;Acc:HGNC:11373]
CAPN5	CAPN5	calpain 5 [Source:HGNC Symbol;Acc:HGNC:1482]
CITED2	CITED2	Cbp/p300 interacting transactivator with Glu/Asp rich carboxy-terminal domain 2 [Source:HGNC Symbol;Acc:HGNC:1987]
KDELR3	KDELR3	KDEL endoplasmic reticulum protein retention receptor 3 [Source:HGNC Symbol;Acc:HGNC:6306]
IDUA	IDUA	alpha-L-iduronidase [Source:HGNC Symbol;Acc:HGNC:5391]
GLRX	GLRX	glutaredoxin [Source:HGNC Symbol;Acc:HGNC:4330]
PLOD2	PLOD2	"procollagen-lysine,2-oxoglutarate 5-dioxygenase 2 [Source:HGNC Symbol;Acc:HGNC:9082]"
IRS2	IRS2	insulin receptor substrate 2 [Source:HGNC Symbol;Acc:HGNC:6126]
PAM	PAM	peptidylglycine alpha-amidating monooxygenase [Source:HGNC Symbol;Acc:HGNC:8596]
SLC16A3	SLC16A3	solute carrier family 16 member 3 [Source:HGNC Symbol;Acc:HGNC:10924]
ENO2	ENO2	enolase 2 [Source:HGNC Symbol;Acc:HGNC:3353]
ANGPTL4	ANGPTL4	angiopoietin like 4 [Source:HGNC Symbol;Acc:HGNC:16039]
GNE	GNE	glucosamine (UDP-N-acetyl)-2-epimerase/N-acetylmannosamine kinase [Source:HGNC Symbol;Acc:HGNC:23657]
GALK1	GALK1	galactokinase 1 [Source:HGNC Symbol;Acc:HGNC:4118]
IL13RA1	IL13RA1	interleukin 13 receptor subunit alpha 1 [Source:HGNC Symbol;Acc:HGNC:5974]
UGP2	UGP2	UDP-glucose pyrophosphorylase 2 [Source:HGNC Symbol;Acc:HGNC:12527]
P4HA1	P4HA1	prolyl 4-hydroxylase subunit alpha 1 [Source:HGNC Symbol;Acc:HGNC:8546]
ZNF292	ZNF292	zinc finger protein 292 [Source:HGNC Symbol;Acc:HGNC:18410]
LHPP	LHPP	phospholysine phosphohistidine inorganic pyrophosphate phosphatase [Source:HGNC Symbol;Acc:HGNC:30042]
DDIT4	DDIT4	DNA damage inducible transcript 4 [Source:HGNC Symbol;Acc:HGNC:24944]
AGRN	AGRN	agrin [Source:HGNC Symbol;Acc:HGNC:329]
PKP2	PKP2	plakophilin 2 [Source:HGNC Symbol;Acc:HGNC:9024]
TGFA	TGFA	transforming growth factor alpha [Source:HGNC Symbol;Acc:HGNC:11765]
VEGFA	VEGFA	vascular endothelial growth factor A [Source:HGNC Symbol;Acc:HGNC:12680]
ELF3	ELF3	E74 like ETS transcription factor 3 [Source:HGNC Symbol;Acc:HGNC:3318]
ARPP19	ARPP19	cAMP regulated phosphoprotein 19 [Source:HGNC Symbol;Acc:HGNC:16967]
HOMER1	HOMER1	homer scaffold protein 1 [Source:HGNC Symbol;Acc:HGNC:17512]
FKBP4	FKBP4	FKBP prolyl isomerase 4 [Source:HGNC Symbol;Acc:HGNC:3720]
CTH	CTH	cystathionine gamma-lyase [Source:HGNC Symbol;Acc:HGNC:2501]
PDK3	PDK3	pyruvate dehydrogenase kinase 3 [Source:HGNC Symbol;Acc:HGNC:8811]
TXN	TXN	thioredoxin [Source:HGNC Symbol;Acc:HGNC:12435]
HK2	HK2	hexokinase 2 [Source:HGNC Symbol;Acc:HGNC:4923]
DSC2	DSC2	desmocollin 2 [Source:HGNC Symbol;Acc:HGNC:3036]
GPR87	GPR87	G protein-coupled receptor 87 [Source:HGNC Symbol;Acc:HGNC:4538]
GOT2	GOT2	glutamic-oxaloacetic transaminase 2 [Source:HGNC Symbol;Acc:HGNC:4433]
ME1	ME1	malic enzyme 1 [Source:HGNC Symbol;Acc:HGNC:6983]
ME2	ME2	malic enzyme 2 [Source:HGNC Symbol;Acc:HGNC:6984]
SLC25A13	SLC25A13	solute carrier family 25 member 13 [Source:HGNC Symbol;Acc:HGNC:10983]
ARTN	ARTN	artemin [Source:HGNC Symbol;Acc:HGNC:727]
GCLC	GCLC	glutamate-cysteine ligase catalytic subunit [Source:HGNC Symbol;Acc:HGNC:4311]
AURKA	AURKA	aurora kinase A [Source:HGNC Symbol;Acc:HGNC:11393]
IER3	IER3	immediate early response 3 [Source:HGNC Symbol;Acc:HGNC:5392]
AGL	AGL	"amylo-alpha-1, 6-glucosidase, 4-alpha-glucanotransferase [Source:HGNC Symbol;Acc:HGNC:321]"
CDK1	CDK1	cyclin dependent kinase 1 [Source:HGNC Symbol;Acc:HGNC:1722]
SOX9	SOX9	SRY-box transcription factor 9 [Source:HGNC Symbol;Acc:HGNC:11204]
STC2	STC2	stanniocalcin 2 [Source:HGNC Symbol;Acc:HGNC:11374]
B3GNT3	B3GNT3	"UDP-GlcNAc:betaGal beta-1,3-N-acetylglucosaminyltransferase 3 [Source:HGNC Symbol;Acc:HGNC:13528]"
TGFBI	TGFBI	transforming growth factor beta induced [Source:HGNC Symbol;Acc:HGNC:11771]

C

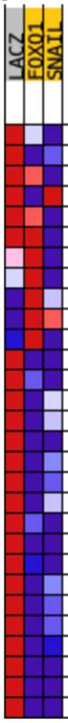

SampleName		
CYB5R3	CYB5R3	cytochrome b5 reductase 3 [Source:HGNC Symbol;Acc:HGNC:2873]
NNT	NNT	nicotinamide nucleotide transhydrogenase [Source:HGNC Symbol;Acc:HGNC:7863]
BCKDHA	BCKDHA	branched chain keto acid dehydrogenase E1 subunit alpha [Source:HGNC Symbol;Acc:HGNC:986]
GLUD1	GLUD1	glutamate dehydrogenase 1 [Source:HGNC Symbol;Acc:HGNC:4335]
IDH2	IDH2	isocitrate dehydrogenase (NADP(+)) 2 [Source:HGNC Symbol;Acc:HGNC:5383]
ETFDH	ETFDH	electron transfer flavoprotein dehydrogenase [Source:HGNC Symbol;Acc:HGNC:3483]
TCIRG1	TCIRG1	"T cell immune regulator 1, ATPase H+ transporting V0 subunit a3 [Source:HGNC Symbol;Acc:HGNC:11647]"
ECH1	ECH1	enoyl-CoA hydratase 1 [Source:HGNC Symbol;Acc:HGNC:3149]
PHYH	PHYH	phytanoyl-CoA 2-hydroxylase [Source:HGNC Symbol;Acc:HGNC:8940]
ALDH6A1	ALDH6A1	aldehyde dehydrogenase 6 family member A1 [Source:HGNC Symbol;Acc:HGNC:7179]
PDK4	PDK4	pyruvate dehydrogenase kinase 4 [Source:HGNC Symbol;Acc:HGNC:8812]
SLC25A4	SLC25A4	solute carrier family 25 member 4 [Source:HGNC Symbol;Acc:HGNC:10990]
IDH3A	IDH3A	isocitrate dehydrogenase (NAD(+)) 3 catalytic subunit alpha [Source:HGNC Symbol;Acc:HGNC:5384]
ATP6V0B	ATP6V0B	ATPase H+ transporting V0 subunit b [Source:HGNC Symbol;Acc:HGNC:861]
CYCS	CYCS	"cytochrome c, somatic [Source:HGNC Symbol;Acc:HGNC:19986]"
TIMM10	TIMM10	translocase of inner mitochondrial membrane 10 [Source:HGNC Symbol;Acc:HGNC:11814]
COX17	COX17	cytochrome c oxidase copper chaperone COX17 [Source:HGNC Symbol;Acc:HGNC:2264]
GRPEL1	GRPEL1	"GrpE like 1, mitochondrial [Source:HGNC Symbol;Acc:HGNC:19696]"
HSPA9	HSPA9	heat shock protein family A (Hsp70) member 9 [Source:HGNC Symbol;Acc:HGNC:5244]
ATP1B1	ATP1B1	ATPase Na+/K+ transporting subunit beta 1 [Source:HGNC Symbol;Acc:HGNC:804]
FXN	FXN	frataxin [Source:HGNC Symbol;Acc:HGNC:3951]
MRPS12	MRPS12	mitochondrial ribosomal protein S12 [Source:HGNC Symbol;Acc:HGNC:10380]
LRPPRC	LRPPRC	leucine rich pentatricopeptide repeat containing [Source:HGNC Symbol;Acc:HGNC:15714]
COX10	COX10	cytochrome c oxidase assembly factor heme A:farnesyltransferase COX10 [Source:HGNC Symbol;Acc:HGNC:2260]
GOT2	GOT2	glutamic-oxaloacetic transaminase 2 [Source:HGNC Symbol;Acc:HGNC:4433]
MRPS15	MRPS15	mitochondrial ribosomal protein S15 [Source:HGNC Symbol;Acc:HGNC:14504]
MRPL35	MRPL35	mitochondrial ribosomal protein L35 [Source:HGNC Symbol;Acc:HGNC:14489]
CYC1	CYC1	cytochrome c1 [Source:HGNC Symbol;Acc:HGNC:2579]
NDUFA9	NDUFA9	NADH:ubiquinone oxidoreductase subunit A9 [Source:HGNC Symbol;Acc:HGNC:7693]

Supplementary Figure 2

Supplementary Figure 2. Gene set enrichment analysis (GSEA) based on 2201 genes commonly dyregulated in HMLE/FOXQ1 and HMLE/SNAL1 cells. A. Hallmark of Epithelial to mesenchymal transition demonstrated by blue pink O'Gram in the space of analyzed gene sets. **B.** Hallmark of glycolysis demonstrated by blue pink O'Gram in the space of analyzed gene sets. **C.** Hallmark of oxidative phosphorylation demonstrated by blue pink O'Gram in the space of analyzed gene sets.

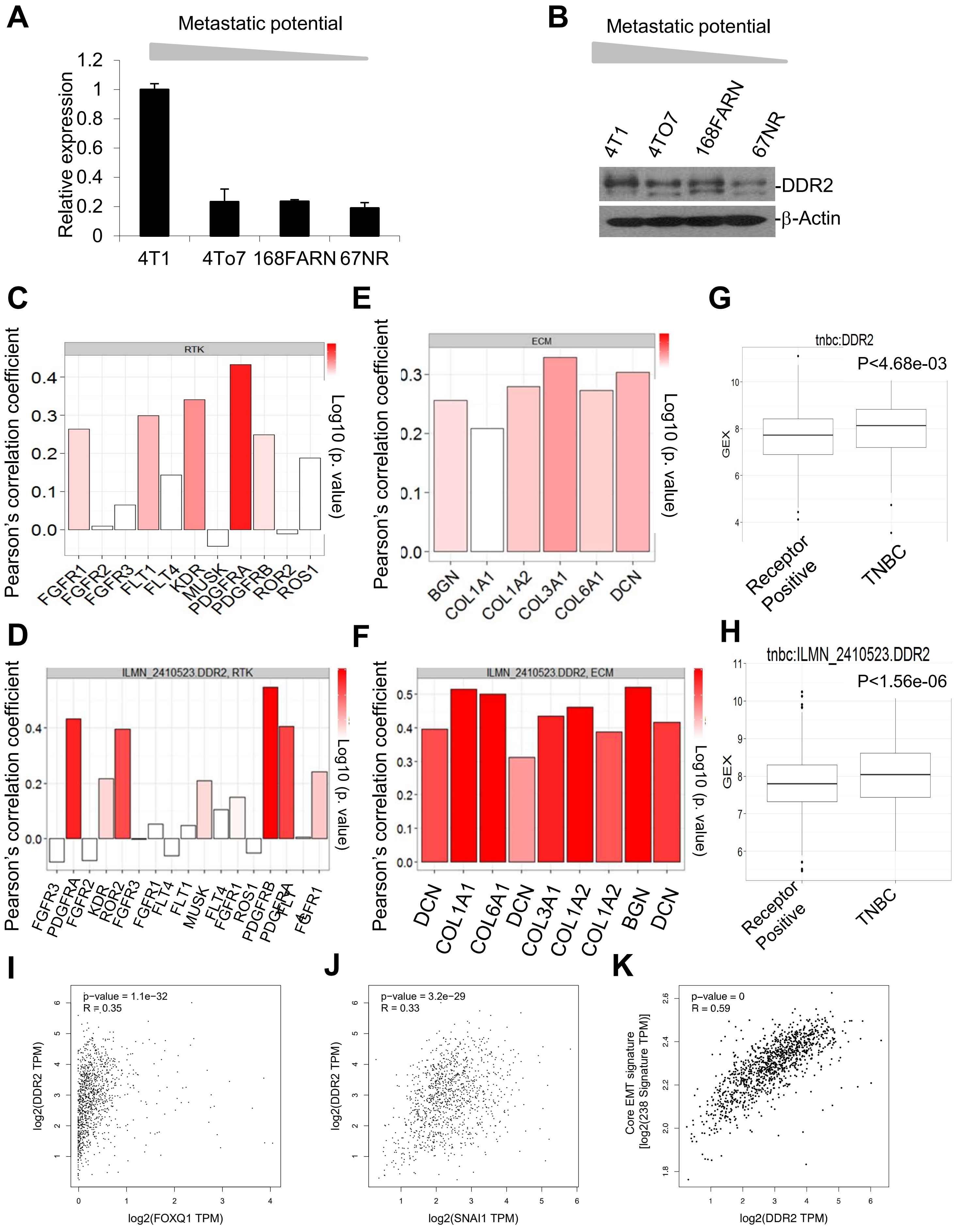

Supplementary Figure 3

Supplementary Figure 3. DDR2 expression in breast cancer. A. DDR2 mRNA expression was examined by q-PCR in a panel of mouse breast cancer cell lines. **B.** DDR2 protein level was detected by western blot analysis in a panel of mouse breast cancer cell lines. **C and D.** Correlation analysis between DDR2 and other receptor tyrosine kinases (RTK) in TCGA (C) and METABRIC (D) databases. Bar plots show Pearson's correlation coefficients between DDR2 and genes denoted on the x-axis. Fill colors represent the significance p-value of correlation coefficients against Ho:ρ = 0. Data is from the Illumina-HiSeq_RNASeqV2 platform. **E and F.** Correlation analysis between DDR2 and extracellular matrix (ECM) in TCGA (E) and METABRIC(F) databases. Bar plots show Pearson's correlation coefficients between DDR2 and genes denoted on the x-axis. Fill colors represent the significance p-value of correlation coefficients against Ho:ρ = 0. Data is from the Illumina-HiSeq_RNASeqV2 platform. **G and H.** Expression of DDR2 showed significantly high in TNBC tumors than in receptor-positive tumors in TCGA (G) and METABRIC(H) databases. Significant p-value from the Kruskal-Wallis test is shown on the right top. **i.** Spearman correlation of FOXQ1 and DDR2 expression in TCGA breast cancer gene expression data. **J.** Spearman correlation of SNAI1 and DDR2 expression in TCGA breast cancer gene expression data. **K.** Spearman correlation of DDR2 expression with the expression of a 238-gene EMT signature (Taube et al. 2010, PMID: 20713713) across TCGA breast cancer samples.

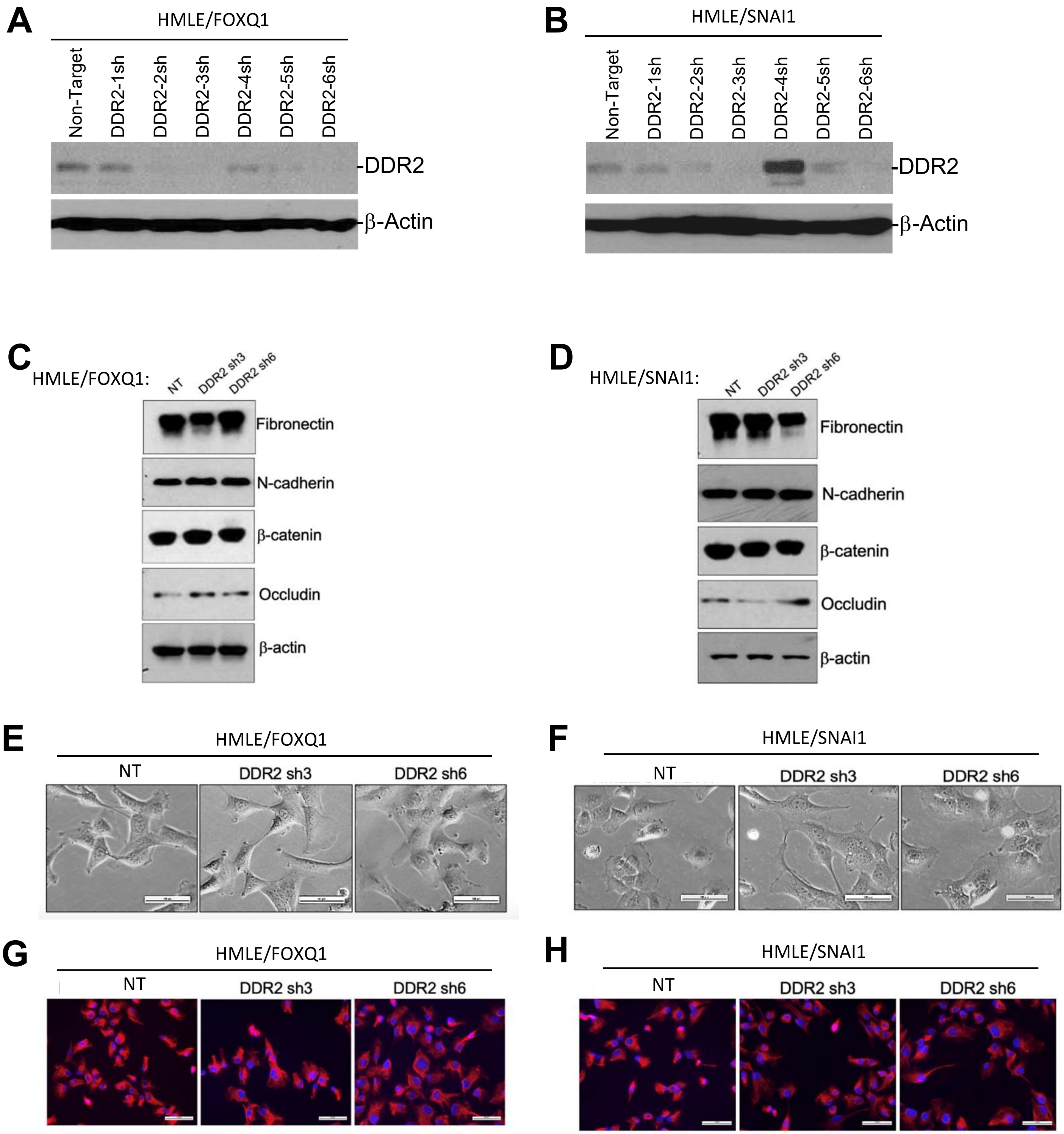

Supplementary Figure 4

Supplementary Figure 4. DDR2 has minimal effect on EMT. A and B. Knockdown of DDR2 in HMLE/FOXQ1 (A) and HMLE/SNAI1 (B) cell models, alongside nontarget (NT) control, were examined by western blot analysis. β-actin was served as a protein loading control. **C and D.** Western blot results show the effect of DDR2 knockdown on EMT marker expression in HMLE/FOXQ1 (C) and HMLE/SNAI1 (D). **E and F.** Representative pictures show no marked morphological change after DDR2 expression in HMLE/FOXQ1 (E) and HMLE/SNAI1 (F) cells. The scale indicates 100 μm. **G and H.** Immunofluorescence analysis of vimentin (red) expression in HMLE/FOXQ1 (G) and HMLE/SNAI1 (H). Blue channel indicates DAPI nuclear stain. The scale suggests 100 μm.

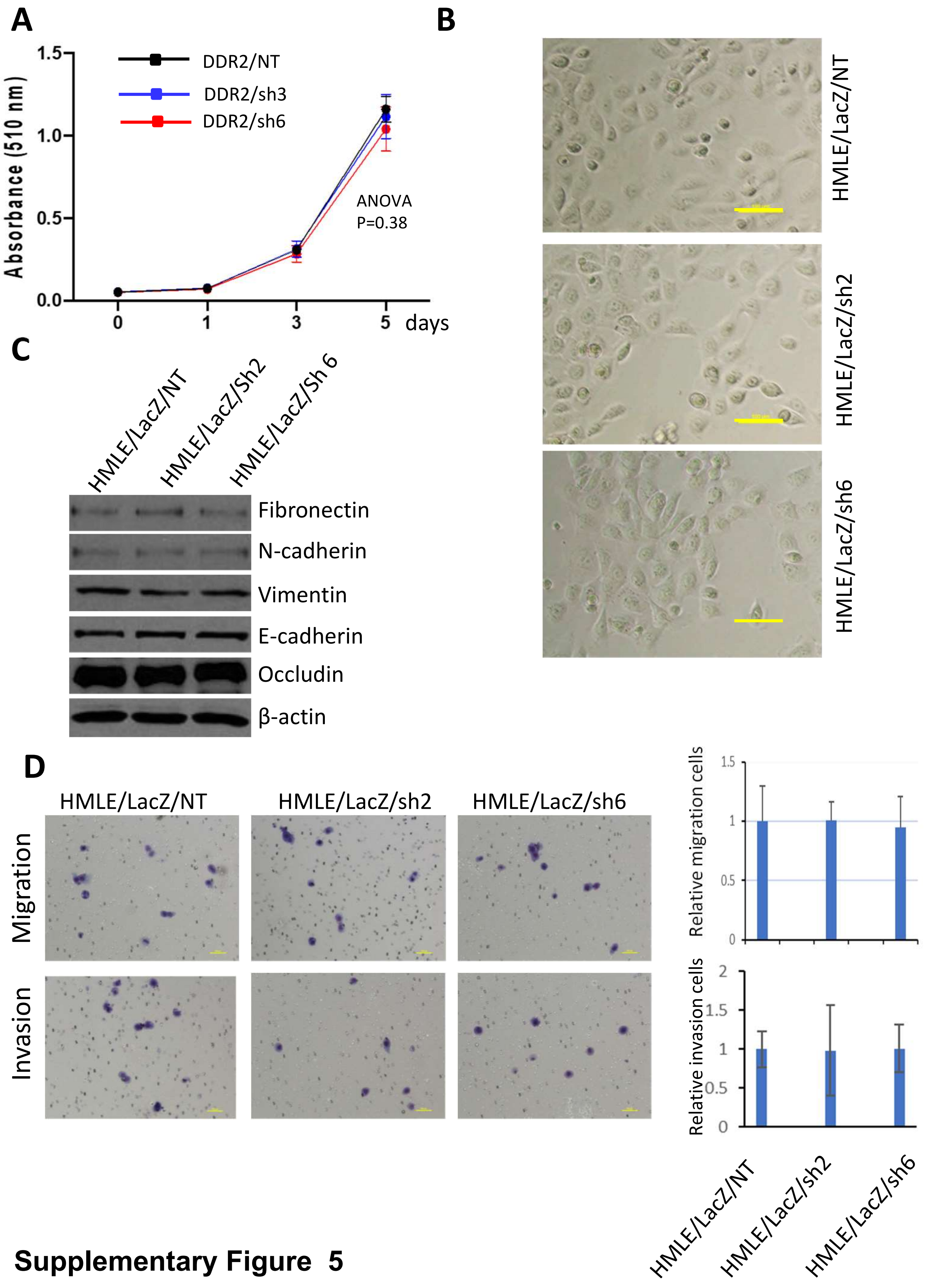

Supplementary Figure 5

Supplementary Figure 5. The effect of DDR2 on cell proliferation, EMT, cell migration, and invasion in HMLE/LacZ control cells. A. Cell proliferation in the HMLE/LacZ cells with or without DDR2 knockdown was measured by Suforhodamine B assay. **B.** Cell morphology of HMLE/LacZ cells with or without DDR2 knockdown remain unchanged. Scale bar:100 μm. **C.** Western blot analysis for mesenchymal markers VIM, FN1 and N-cadherin and epithelial marker CDH1 and Occludin was performed in the HMLE/LacZ with or without DDR2 knockdown. **D.** Cell migration and invasion assay was performed in the HMLE/LACZ with or without DDR2 knockdown.

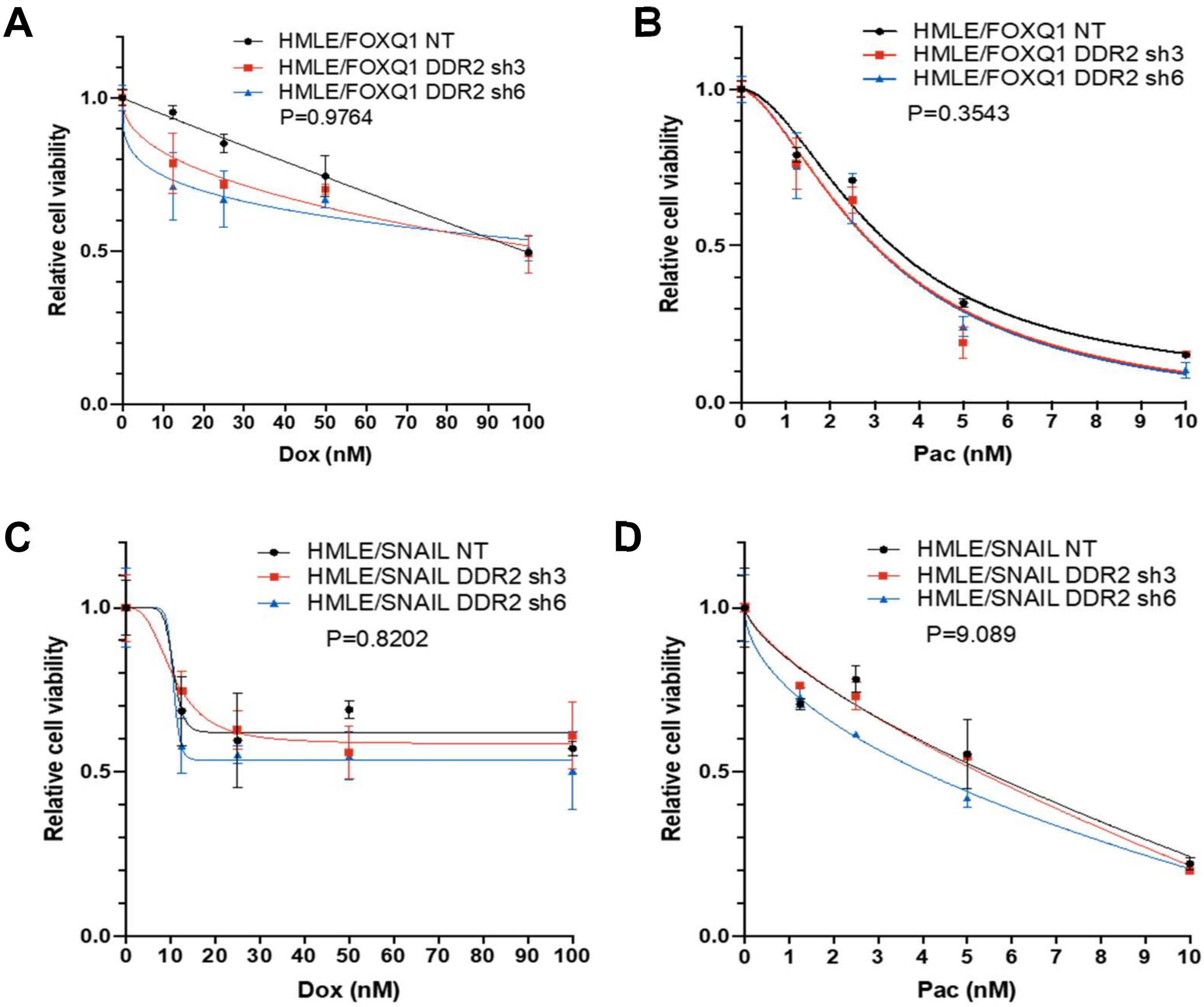

Supplementary Figure 6

Supplementary Figure 6. The effect of DDR2 on stemness properties in EMT cell models. A and B. HMLE/FOXQ1 cells with DDR2 knockdown or NT control were treated with indicated doses of doxorubicin (A) or paclitaxel (B) as indicated for 24 hours. **C and D.** HMLE/SNAI1 cells with DDR2 knockdown or NT control were treated with indicated doses of doxorubicin (C) or paclitaxel (D) as indicated for 24 hours. For all panels, cell survival was analyzed by an MTT assay, and results are graphed relative to solvent control. No significant difference was shown by statistical analysis.

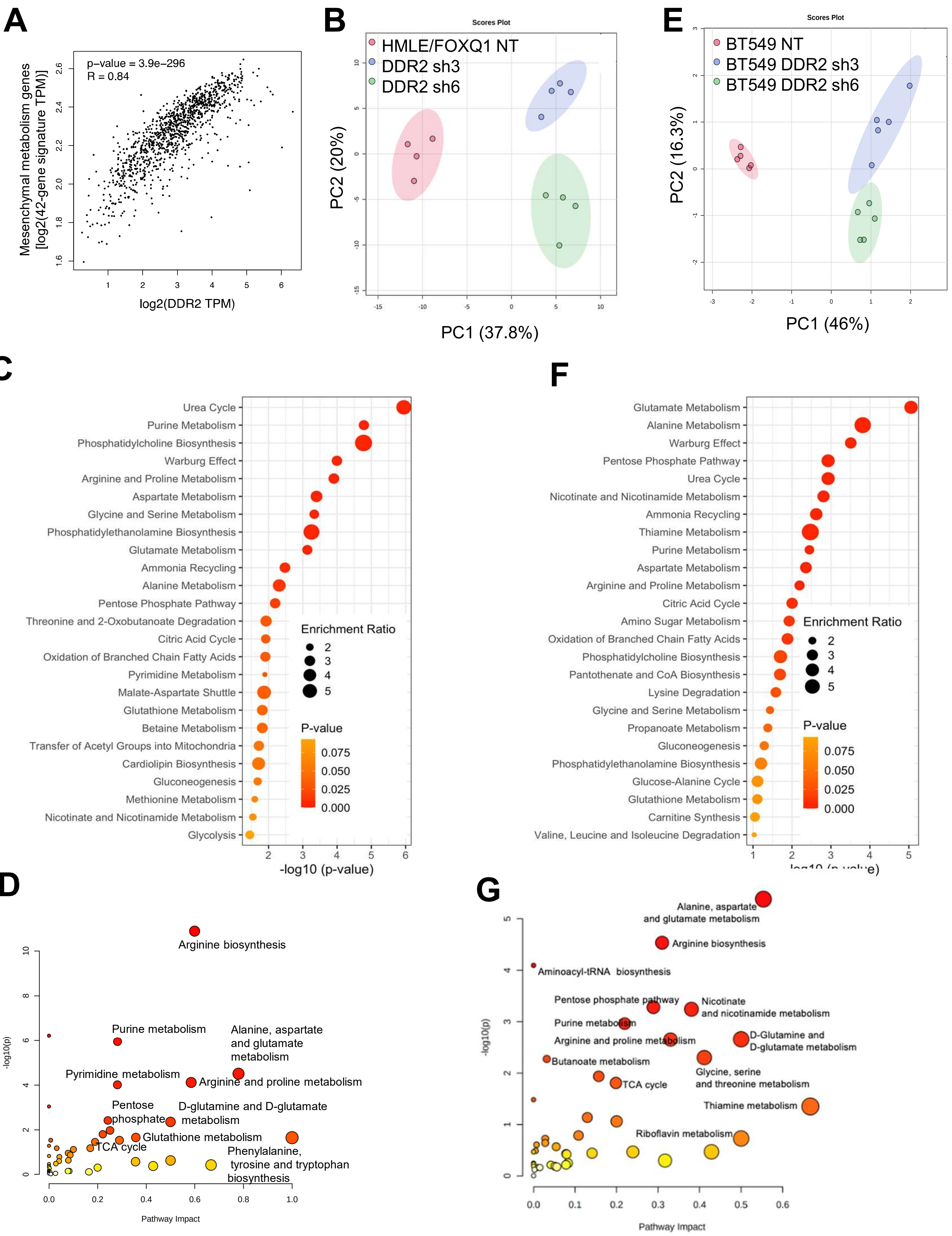

Supplementary Figure 7

Supplementary Figure 7. The effect of DDR2 knockdown on cellular metabolism

(A) Spearman correlation of DDR2 expression with the expression of a 42 mesenchymal metabolism gene signature across TCGA breast cancer samples. (B) Principal component analysis of the metabolite profiles of the HMLE/FOXQ1 NT and DDR2 knockdown cell lines. (C) Metabolite functional annotation of the 106 metabolites affected by DDR2 knockdown in HMLE/FOXQ1 cells using SMPDB database functional annotation, top 25 enriched terms determined by overrepresentation analysis are shown. (D) Pathway analysis of metabolites altered upon DDR2 knockdown in HMLE/FOXQ1 cells was conducted using hypergeometric testing and KEGG pathway annotations. (E) Principal component analysis based on the metabolic profiles of BT549 cells with stable shRNA knockdown of DDR2 of NT control. (F) Metabolite functional enrichment analysis of the 82 metabolites found to be significantly altered by DDR2 knockdown in BT549 cells using SMPDB database functional annotation system; top 25 enriched pathways are shown. (G) Results of KEGG pathway analysis of significantly altered metabolites in the BT549 DDR2 knockdown models performed by hypergeometric testing.

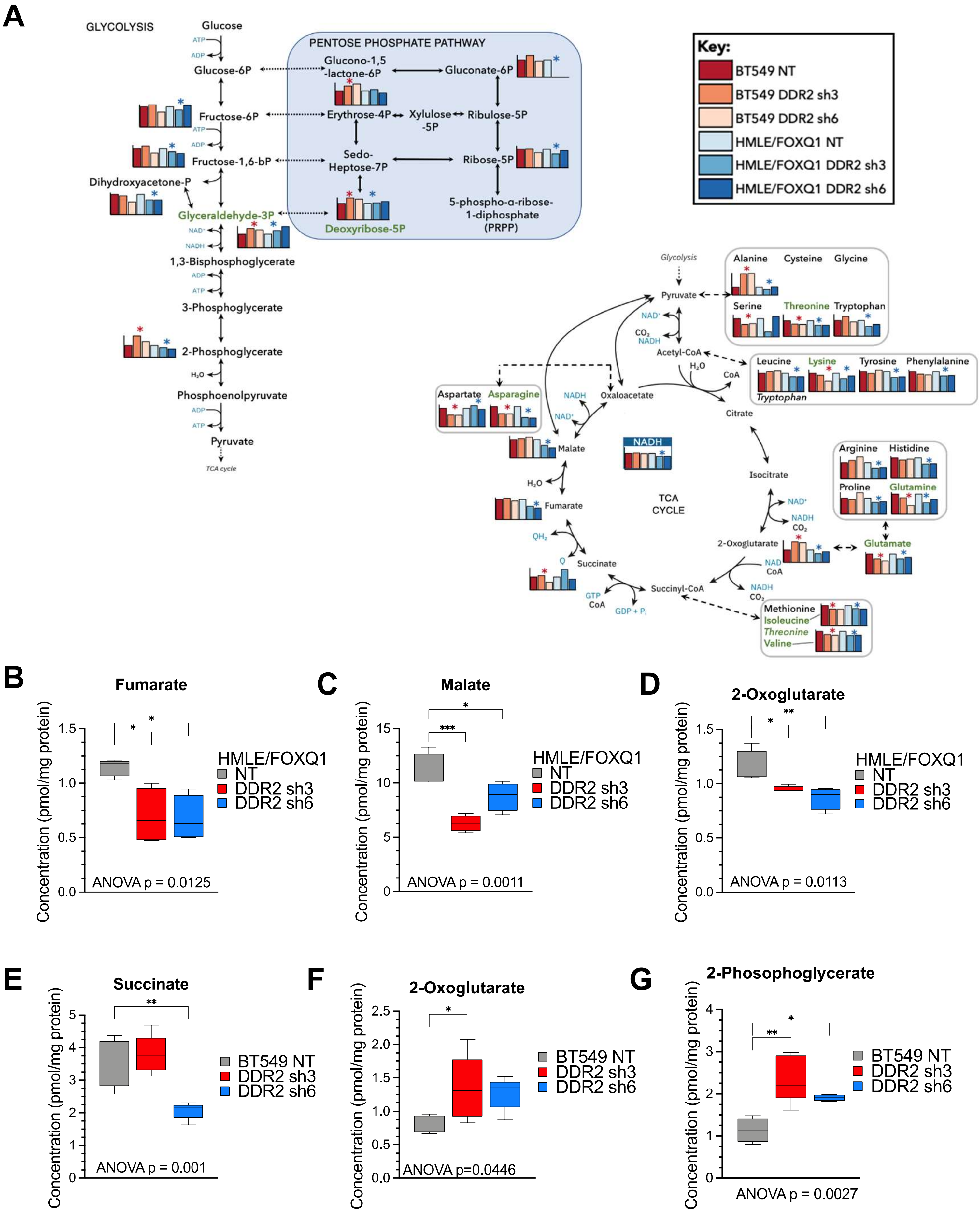

Supplementary Figure 8

Supplementary Figure 8. Model-specific metabolites changes in HMLE/FOXQ1 cells (EMT) and BT549 cells (TNBC) within the TCA .
(A) Overview of metabolic changes to central carbon metabolism, adapted from MetaboMAPS (metabomaps.brenda-enzymes.org). Bar graphs depict relative metabolite abundance normalized to match NT control sample. Star indicates p <0.05. Green text highlights metabolic changes upon DDR2 shRNA knockdown that were significantly altered in both BT549 and HMLE/FOXQ1 cell models. (B-D) Box and whisker plot of the metabolite abundance from HMLE/FOXQ1 DDR2 knockdown cells and NT control by mass spectrometry metabolomics. HMLE/FOXQ1 DDR2 knockdown cells display a decrease in the abundance of TCA metabolites fumarate (B) , malate (C), and 2-oxoglutarate/alpha-ketoglutarate (D). (E-G) Box and whisker plot of the metabolite abundance from BT549 DDR2 knockdown cells and NT control. DDR2 knockdown cells also had a differential abundance of TCA metabolites succinate (E), 2-oxoglutarate/alpha-ketoglutarate (F), and 2-phosphoglycerate (G).

Supplementary Table 1: Twenty-three metabolites commonly dysregulated by DDR2 knockdown in HMLE/FOXQ1 and BT549 cells

	HMLE/FOXQ1				BT549			
**Metabolite**	**Fold change**	**log2(FC)**	**p-value**	**-log10(p-value)**	**Fold change**	**log2(FC)**	**p-value**	**-log10(p-value)**
**L-Aspartic acid**	0.44452	-1.1697	4.28E-08	7.369	0.63415	-0.65711	0.028419	1.5464
**L-Threonine**	0.71959	-0.47476	4.91E-08	7.3085	0.7708	-0.37556	0.073809	1.1319
**4-Hydroxyproline**	0.57627	-0.79519	2.34E-06	5.6313	0.70229	-0.50986	6.70E-05	4.1738
**L-Valine**	0.79782	-0.32587	1.41E-05	4.8523	0.82715	-0.27379	0.0017302	2.7619
**N-Methyl-D-aspartic acid**	0.81067	-0.30281	2.24E-05	4.6496	0.72703	-0.45992	0.00053897	3.2684
**L-Glutamic acid**	0.78601	-0.34739	2.49E-05	4.604	0.69971	-0.51517	0.00033961	3.469
**1-Methylhistidine**	0.67165	-0.57421	6.41E-05	4.1932	0.79041	-0.33933	0.071561	1.1453
**L-Isoleucine**	0.83068	-0.26764	6.51E-05	4.1863	0.81092	-0.30236	0.019652	1.7066
**Pantothenic acid**	0.72594	-0.46209	6.76E-05	4.1698	0.63525	-0.65461	0.00086976	3.0606
**L-Kynurenine**	0.4898	-1.0297	0.00025911	3.5865	0.37051	-1.4324	4.66E-05	4.332
**L-Glutamine**	0.64615	-0.63005	0.00026027	3.5846	0.62096	-0.68743	0.00557	2.2541
**Pyridoxamine**	0.77094	-0.3753	0.00064772	3.1886	0.75196	-0.41127	0.056318	1.2494
**dCMP**	0.57877	-0.78893	0.0015274	2.8161	0.53394	-0.90525	0.064202	1.1925
**L-Palmitoylcarnitine**	0.36047	-1.4721	0.0090877	2.0415	0.76895	-0.37904	0.020709	1.6838
**Biotin**	0.74503	-0.42462	0.0091419	2.039	0.6564	-0.60735	0.002278	2.6425
**Phenylacetylglycine**	0.81931	-0.28752	0.011842	1.9266	0.78145	-0.35577	0.05291	1.2765
**Uridine diphosphate glucose**	0.82245	-0.282	0.025303	1.5968	0.7405	-0.43343	0.047906	1.3196
**L-Lysine**	0.77171	-0.37388	0.05371	1.2699	0.72651	-0.46094	0.010505	1.9786
**L-Dihydroorotic acid**	0.66296	-0.59301	0.062449	1.2045	0.79971	-0.32244	0.065206	1.1857
**Citicoline**	2.6307	1.3954	0.072735	1.1383	2.2954	1.1988	0.010291	1.9876
**Sorbitol**	2.262	1.1776	0.00062454	3.2044	1.5866	0.66597	0.045173	1.3451
**D-Glyceraldehyde 3-phosphate**	1.3998	0.48519	0.068537	1.1641	1.3511	0.43414	0.056451	1.2483
**Xanthosine**	1.3271	0.40831	0.0026529	2.5763	9.2131	3.2037	8.19E-13	12.087
