# Supplementary material for: DDR2 Coordinates EMT and Metabolic Reprogramming as a Shared Effector of FOXQ1 and SNAI1
Source: Cancer Res Commun. 2022 Nov 9;2(11):1388–403. doi: 10.1158/2767-9764.CRC-22-0013 (PMC9881645; doi:10.1158/2767-9764.CRC-22-0013)
Supplement: Supplementary Primers and shRNA Information — Information of PCR primers for all gene tested and information for shRNA sequences for targeting several genes. [file crc-22-0013-s02.pdf]

# Supplementary Primers and shRNA information

## A. qPCR primers information

1. hFoxq1. QF:aacgactgcttcgtcaagt
   QR:gcatccagtagttgtccttgc

2. hTwist1: QF: tcggtctggaggatggagggg
   QR: aatgacatctaggtctccggccc

3. hSNAI1. QF: tcaggaagccctccgacccc
   QR: agggctgctggaaggtaaactctgg

4. hZeb1. QF: tgacctgccaacagaccagaca
   QR: cctttcctgtgtcatcctcccagc

5. hZeb2. QF:tggtcacacacaagcccagggac
   QR:acttgcgattacctgctccttggg

6. hFoxC1. QF: ggtgcgggagatgttcgagtca
   QR: actggctggaagggaaggcca

7. hFoxC2. QF: cgagccgtctcggaagcagc
   QR: gctcgctcaggtagggcacc

8. hRor2 QF: ctctcagtgtcccggacttc
   QR: ctgcccatcaaggggtccta

9. hMUSK QF: cagcctcgggacagcatatt
   QR: acattgtgggattcaggagcc

10. hMet QF: cagctgacttgctgagagga
    QR: aggtttatctttcggtgccc

11. hEGFR QF: ggccgacagctatgagatgg
    QR: ttccgttacacactttgcgg

12. hRon. QF: ctctggggaccaggttttcc
    QR: aatgccatgcccttaggcaa

13. hErbB2. QF:ttttggaccggaggctgacc
    QR: gagaggtcaggtttcacaccg

14. hErbB3. QF: tccttcctgcagtggattcg
    QR: ggaggttgggcaatggtaga

15. hErbB4. QF: cacccaagggtgtaacggtc
    QR: tgcaatcaggggagttctagc

16. hMer. QF: ttgaagcagcccgaagactg
    QR: gaaaaggtggggcggtctaa

17. hTie1. QF: ctgtgagaagtcagaccgga
    QR: cggggcatcgtctctaagtt

18. hInsR. QF: taccccggagaggtgtgtc
    QR: gtgtccttcgatgacagagca

19. hTie2: QF:agccttccaaaacgtgaggg
    QR: tgggttgtttttgaccttcctg

20. hIGF1R. QF: cgctctggccgacgagt
QR: tcgatcaccgtgcagttctcc

21. hEphA1-8. QF: ttctctgcctcagggactcag
QR: acactgggaacacctcacact

22. hInsRR. QF: ttgtgcaccgagatctagca
QR: tcccgaagtccccgatctt

23. hEphA10. QF: actgccaagtaatgggtggg
QR: ccagcacattgcacacttgg

24. hCSF1R. QF: cttccaaaacacggggacct
QR: cgggcagggtctttgacata

25. hEphB1-4. QF: agtctccagcaagagtccct
QR: cttggtgcatgatgggaacg

26. hFlt3. QF: aacggccatccttccctaat
QR: cattccgaaacacggccatc

27. hVEGFR1. QF: gaagggagctcgtcattccc
QR: tgcgttttccatcagggatca

28. hEphB6. QF: aatacagcagcccaggactc
QR: caagttctcggatggcctga

29. hRet. QF: cacgggcgatggcgaag
QR:tagaggcccaatgccactttg

30. hVEGFR2. QF: aggaggaagtatgtgacccca
QR: ccggctctttcgcttactgt

31. hVEGFR3. QF: ccgggacatctacaaagaccc
QR: ttcaggggccatccacttca

32. hFGFR1. QF: ccttgacctccaaccaggagta
QR:cacgtagagctccgggtgtc

33. hFGFR2. QF:ggtgtcatgcacctaccagc
QR:tctggctgctaaatctcgatgaa

34. hFGFR3. QF:tgaatgcctcccacgaggac
QR:atggagcgtctgtcacccg

35. hFGFR4. QF:ccttgacctccagcaacgat
QR:tgtgtccagtagggtgcttg

36. hRyk, QF: ccagttcgttggatggctct
QR: agtcatgagttcccacagcg

37. hDDR1. QF: ctggagggatggactcctgt
QR: gttgaggtacacggcctcag

38. hDDR2. QF:gagccatccaggctgatacg
QR:ggctggactggcttcacaa

39. hRos. QF: ctttcggtgctgtttggctc
QR: actgatcaggatgacattggct

40. hLMR1. QF: gtgtacgtcctgccactcac.

QR: gacttgaggagctgcactga

41. hLMR2. QF: tccctacctcctggtgtttga
QR: cagcatggtctgtgagtccc

42. hLMR3. QF:ggcaagtgctgtggttgtgt
QR:caggcatcttgtcgaggatgg

43. hTrkA. QF: ctgccttcatggacaaccct
QR: ttagtgtccaccggcgagaa

44. hTrkB. QF: tctgctcacttcatgggctg
QR: cgctgcagttccataatcttca

45. hTrkC. QF: cacagccaaccagaccatca
QR: tgggactcacttcgtcaaacaa

46. hALK. QF: gtgctacagtgaccagtgct
QR: ccacgaatgagccaggacat

47. hLTK. QF: ccctggccagtatctgatctc
QR: gattgctgagacgaagacgc

48. hSTYK1. QF: cctgtcattatttcccctccga
QR: tgcagcccagtgaaattgga

49. hRor1. QF: gtttgtcaagtttggccccc
QR: tgcacatgcaatccctctgt

**B. shRNA information**

| Target genes | Clone ID | Mature Antisense Sequence |
|---|---|---|
| DDR2 Sh3 | TRCN0000001419 | AACATGCTAGAATCACTTGGC |
| DDR2 Sh6 | TRCN0000121175 | ATAACCTGTTCATCTGACAGC |
| FOXQ1 Sh1 | TRCN0000017923 | TACTCGTTGATCTCCGCCAGC |
| FOXQ1 Sh2 | TRCN0000017924 | AACTTGCCCATGAGGTACTCG |
| SNAI1 Sh3 | TRCN0000063820 | TGTAGTTAGGCTTCCGATTGG |
| SNAI 1 Sh5 | TRCN0000063822 | AACTCTGGATTAGAGTCCTGC |
